# Targeting the YAP‐BST2 Axis Overcomes Intrinsic Anti‐PD‐1 Resistance in Metastatic Gastric Cancer

**DOI:** 10.1002/advs.77708

**Published:** 2026-09-21

**Authors:** Weihong Zhang, Shilong Wang, Meng Wang, Ruixian Yu, Jingwu Yue, Lin Shao, Hui Zhang, Mengwen Zhu, Luyang Tian, Shuting Cheng, Weimin Qin, Yang Tang, Yi Han, Wenjia Wang, Liwei An, Yan Meng, Shi Jiao, Zhaocai Zhou

**Affiliations:** ^1^ State Key Laboratory of Genetics and Development of Complex Phenotypes School of Life Sciences Zhongshan Hospital Fudan University Shanghai China; ^2^ School of Medicine Shanghai Tenth People's Hospital Tongji University Cancer Center Tongji University Shanghai China; ^3^ Department of Gastroenterology School of Medicine Shanghai Ninth People's Hospital Shanghai Jiao Tong University Shanghai China; ^4^ Collaborative Innovation Center for Cancer Personalized Medicine School of Public Health Nanjing Medical University Nanjing China; ^5^ Tianfu Jincheng Laboratory Chengdu China

**Keywords:** gastric cancer, genetic engineered mouse model, immune evasion, immunotherapy resistance, metastatic GC, YAP‐BST2

## Abstract

Intrinsic resistance to anti‐PD‐1 immunotherapy remains a major obstacle in treating metastatic gastric cancer (GC), particularly in tumors harboring concurrent YAP hyperactivation and TP53 loss. Here, using a genetically engineered mouse model with conditional YAP hyperactivation and Tp53 deletion in gastric Atp4b^+^ cells (AYP), we show this “double‐hit” alone suffices to recapitulate human refractory GC, including histopathological heterogeneity, multi‐organ metastasis, and intrinsic PD‐1 resistance. We identified BST2 as a direct YAP‐TEAD transcriptional target. In human GC, BST2‐high tumor correlates with poor anti‐PD‐1 response. Mechanistically, tumor cell‐derived BST2 engages the inhibitory receptor PIRA2 on neutrophils and liver Kupffer cells, instructing an immunosuppressive, pro‐metastatic phenotype that inhibits T cells antitumor response and confers PD‐1 resistance. Therapeutically, dual BST2/PD‐1 blockade in the AYP model suppresses primary tumor growth and eradicates established liver metastases. Thus, YAP activation, cooperating with TP53 loss, fuels metastatic GC and immunotherapy resistance via BST2 induction.

## Graphical abstract

Concurrent YAP hyperactivation and Tp53 loss in Atp4b‐expressed parietal cell lineage drives gastric cancer initiation, multi‐organ metastasis, and intrinsic resistance to anti‐PD‐1 therapy. BST2 emerges as a direct YAP‐TEAD transcriptional target that engages PIRA2 on myeloid cells to establish an immunosuppressive microenvironment. Dual BST2/PD‐1 blockade suppresses gastric cancer progression, offering a promising therapeutic avenue.

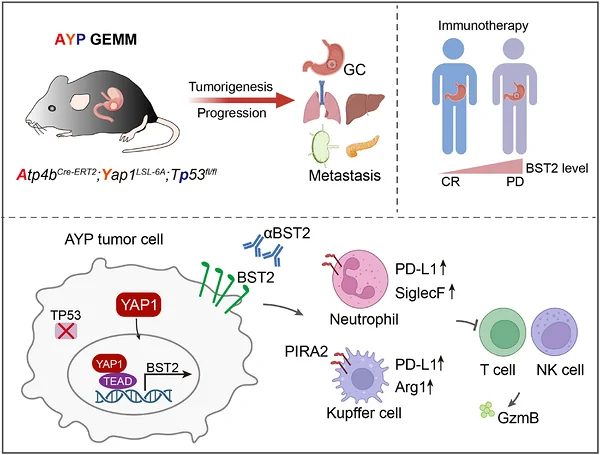

## 1 Introduction

Gastric cancer (GC) is a molecularly and histopathologically heterogeneous disease with a high propensity for metastasis, which, coupled with frequent therapeutic resistance, leads to poor patient outcomes. While immune checkpoint blockade (ICB), particularly anti‐PD‐1/PD‐L1 therapy, has revolutionized the management of multiple cancers, its efficacy in GC remains limited, with response rates below 30% and a significant proportion of patients exhibiting intrinsic resistance [1, 2]. This underscores an urgent need to delineate the molecular drivers of immunosuppression in GC.

Genomically, a substantial subset of GCs, particularly those with chromosomal instability (CIN), harbor concurrent alterations in two key pathways: loss of the tumor suppressor TP53 and hyperactivation of the oncogenic transcriptional co‐activator YAP [3, 4, 5, 6, 7]. YAP activation, a hallmark of Hippo pathway dysregulation, is implicated in gastric tumorigenesis, metastasis, and therapy resistance [8, 9, 10]. Clinically, the co‐occurrence of YAP hyperactivation and TP53 loss correlates with aggressive, refractory disease. However, whether this genetic combination plays a causal role in driving the lethal phenotype, especially intrinsic immunotherapy resistance, and through what mechanism, remains a critical unanswered question.

Existing genetically engineered mouse models (GEMMs) of GC have provided valuable insights but often fail to fully capture the complexity of human refractory disease, including its profound heterogeneity, metastatic cascade, and intrinsic resistance to ICB [11, 12, 13, 14, 15, 16, 17, 18, 19, 20]. To address this gap and directly test the pathological synergy between YAP and TP53, we developed a novel GEMM featuring conditional activation of a stable YAP mutant (*Yap1^6A/6A^*) and deletion of Tp53 specifically in the gastric Atp4b‐expressed parietal cell lineage (AYP model). We found that this “double‐hit” genetic configuration is necessary and sufficient to drive aggressive, metastatic GC that mirrors the histopathological spectrum and intrinsic anti‐PD‐1 resistance seen in patients. Using this model, we performed an unbiased discovery to reveal bone marrow stromal cell antigen 2 (BST2) as a pivotal downstream effector. We establish BST2 not only as a direct YAP target gene, but also as a novel immune checkpoint that engages PIRA2 on innate immune cells. This axis educates both circulating neutrophils and Kupffer cells to foster an immunosuppressive microenvironment, enabling metastasis and subverting anti‐PD‐1 therapy. Importantly, combinatorial targeting of BST2 and PD‐1 potently reversed resistance and eradicated metastatic tumors. Our findings unveil a YAP‐BST2‐driven resistance pathway and propose a readily translatable combinatorial immunotherapy strategy for a refractory subset of GC.

## 2 Results

### 2.1 Concurrent YAP Hyperactivation and TP53 Loss in Parietal Cell Lineage Drives Aggressive and Metastatic GC

**FIGURE 1:**
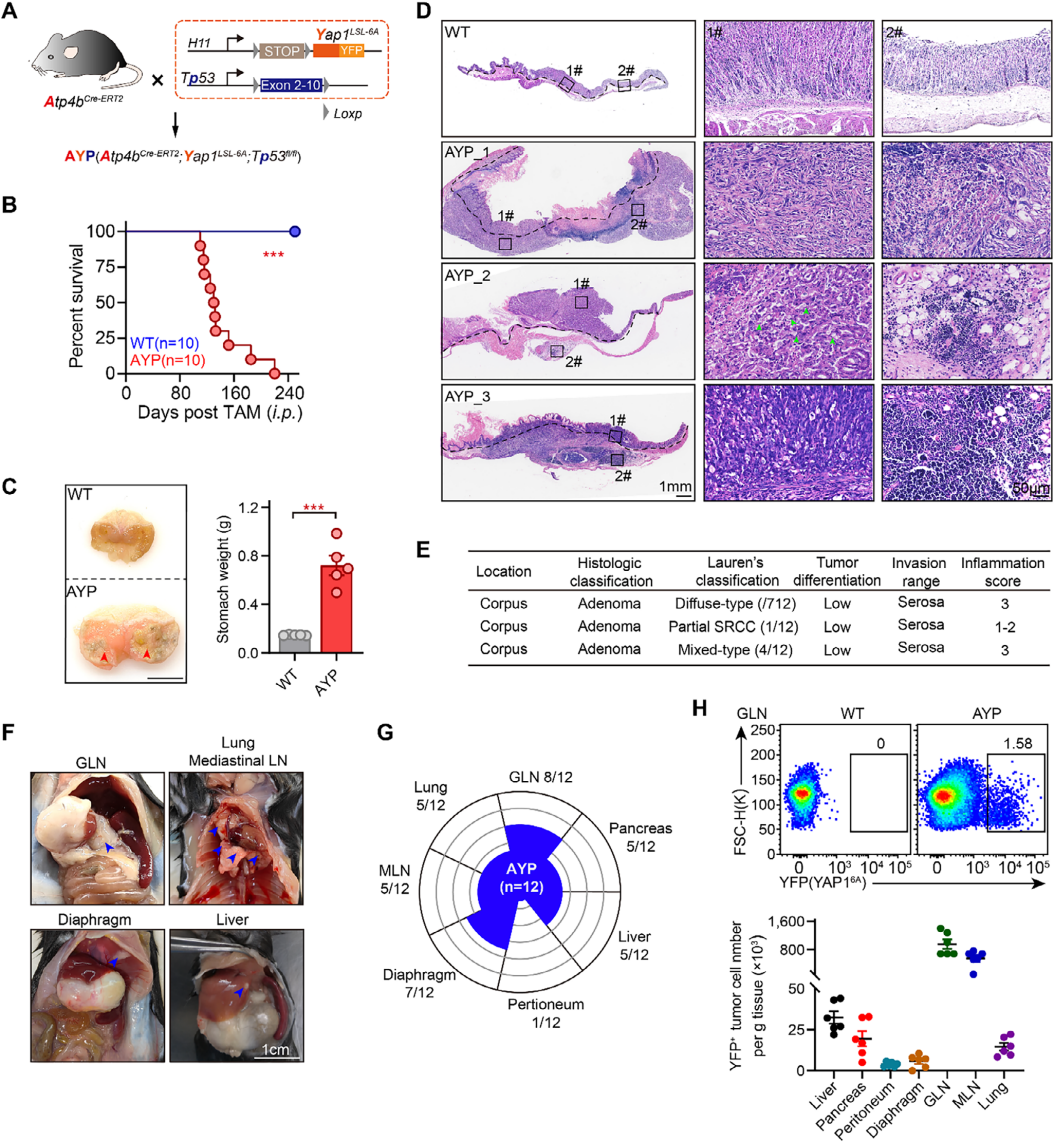
AYP mice generate aggressive GC. (A) Schematic diagram of genetically engineered mouse models (GEMM) of gastric cancer by genetic manipulation within Atp4b‐expressed cells. A CAG‐LSL‐Yap1(6A, S46A/T68A/S94A/S112A/S149A/ S382A)‐YFP‐WPRE‐polyA cassette was inserted into the H11 locus. YFP is expressed as an in‑frame fusion protein with YAP1(6A), without an intervening linker sequence. To induce gastric tumorigenesis, AYP mice were administrated with tamoxifen (100 mg/kg) intraperitoneally for 3 consecutive days at 8 weeks old. (B) Survival of AYP and WT control mice after GC induction (*n* = 10 mice per group), day 0 indicated the time of the first tamoxifen injection. (C) Macroscopic picture showed gastric tumorigenesis of AYP and WT control mice at day 90 after tamoxifen induction, red arrow indicated the primary location of gastric lesions. The stomach weights w ere measured (*n* = 5 mice per group). Scale bar, 1 cm. (D) H&E staining of gastric sections from AYP and WT control mice, representative pictures showed pathological features of GC with diffuse‐type (represented by AYP_1), signet‐ring cell carcinoma (SRCC, represented by AYP_2), and mixed‐type (represented by AYP_3) from AYP mice, and immune cells aggregates in the tumor area. Green arrow indicated the SRCC tumor cells. (E) Summary of GC primary location, histologic and Lauren’ classification, tumor differentiation state, invasive range and inflammation score of the gastric lesions from AYP mice, these parameters were reviewed and scored by two pathologists based on the acquired images of stained primary GC tissue sections in a double‐blind manner. Numbers in the bracket indicated the incidence of gastric cancer with diffuse‐type, SRCC, and mixed type pathological features in AYP mice at 4 months after tamoxifen induction. (F) Representative macroscopic picture showed GC metastasis to gastric lymph node (GLN), diaphragm, liver, lung, and mediastinal lymph node (MLN) from AYP mice, blue arrow indicated the metastatic lesions in these organs. (G) The petal graph showed the metastatic incidence of AYP mice at 4 months after GC induction (*n* = 12 mice). Each radius indicated the metastatic frequencies of the indicated organs, the outermost ring corresponds to 100%. (H) Flow cytometry analysis of metastatic tumors cells (YAP1^6A^‐YFP) in AYP mice. Metastatic tumor cells number were normalized by tissue weight (*n* = 6 mice). Data represent the means ± SEM. Log‐rank (Mantel‐Cox) test was performed for survival comparison (B). Unpaired two‐tailed Student's *t*‐test was used for comparisons (C). ^***^, *p* < 0.001. See also Figure S1.

YAP is a Hippo pathway co‑activator regulated by MST/LATS‑mediated phosphorylation [21, 22] (Figure S1A). YAP1 is amplified in ∼18% of human GC^3^ (Figure S1B), and Hippo‐YAP signaling pathway which has been extensively investigated as a major driving force for gastric tumorigenesis [9, 10, 23], is modulated by diverse extrinsic factors [21]. We found that infection with *Streptococcus anginosus*, a bacterium recently implicated in gastric cancer pathology [24, 25, 26], potently activates this pathway (Figure S1C). To dissect the pathological consequences of YAP hyperactivation in the context of TP53 loss, we generated a GEMM carrying conditional alleles of constitutively active YAP1 (*LSL*‐*Yap1^6A/6A^‐YFP*, 6A mutation, S46A/T68A/S94A/S112A/S149A/S382A, prevents phosphorylation and degradation of YAP1) and floxed Tp53 in gastric Atp4b‐expressed parietal cell lineage (including parietal cell progenitors, pre‐parietal cells and mature parietal cells, Figure S1D): *Atp4b^Cre‐ERT2^*;*LSL*‐*Yap1^6A/6A^*; *Tp53^fl/fl^* (hereafter AYP) (Figure 1A). Following tamoxifen induction, AYP mice developed invasive gastric adenocarcinoma within 2–3 months, with increased stomach weights and a median survival of 131 days (Figure 1B,C). No tumors were formed in the AYP mice without tamoxifen induction (Figure S1E). In addition, neither AY (*Atp4b^Cre‐ERT2^;LSL‐Yap1^6A/6A^*, YAP activation alone) nor AP (*Atp4b^Cre‐ERT2^;Tp53^fl/fl^*, Tp53 loss alone) mice developed gastric cancer even 10 months post tamoxifen induction (Figure S1F); suggesting that YAP activation and p53 loss cooperated to drive gastric cancer initiation. Histopathological analysis revealed three distinct subtypes of AYP GC, well‐differentiated (intestinal type), poorly differentiated with signet ring cells (diffuse type), and mixed or indeterminate forms, all accompanied by dense immune infiltration (Figure 1D,E).

By 3–4 months post induction, the majority of AYP mice developed multi‐organ metastases, involving gastric lymph node (GLN, 8/12), diaphragm (7/12), liver (5/12), pancreas (5/12), mediastinal lymph node (5/12), and lung (5/12) (Figure 1F,G). Metastatic lesions exhibited poorly differentiated histology with atypical nuclei and aberrant gland formation (Figure S1G,H). In addition, YFP^+^ cells arise in the metastatic lesions of AYP mice (Figures 1H and S1I,J), suggesting the dissemination of Atp4b‐expressed tumor cells. These results collectively demonstrate that concurrent YAP hyperactivation and TP53 loss in the parietal cell lineage is sufficient to drive both primary gastric tumorigenesis and aggressive systemic metastasis.

### 2.2 AYP GC Mice Exhibit Resistance to Anti‐PD‐1 Immunotherapy

**FIGURE 2:**
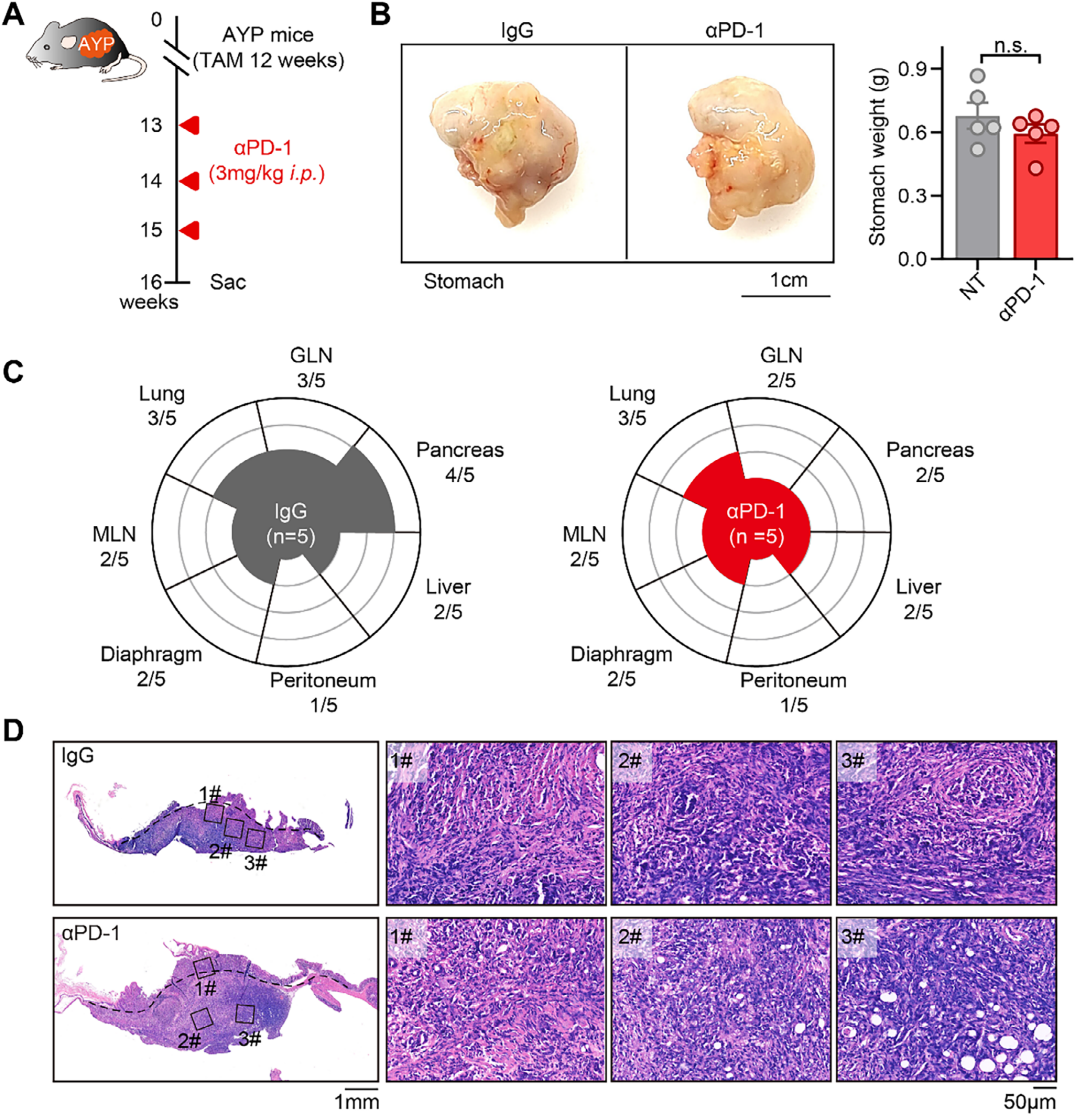
AYP mice show resistance to anti‐PD‐1 therapy. (A) Schematic diagram of anti‐PD‐1 antibody treatment of AYP mice. AYP mice (12 weeks after TAM injection) were intraperitoneally injected with anti‐PD‐1 antibodies (3 mg/kg) weekly for 3 weeks, and then sacrificed to examine gastric pathological and immune features. (B) Macroscopic picture showed the gastric tissues from AYP mice with or without anti‐PD‐1 antibody treatment, the stomach weights ware measured (*n* = 5 mice per group). Scale bar, 1 cm. (C) Petal graphs showed metastatic incidence of anti‐PD‐1 antibody‐treated and untreated mice (*n* = 5 mice per group). Each radius indicated the metastatic frequencies of indicated organs, the outermost ring corresponds to 100%. (D) H&E staining of gastric sections from AYP mice after anti‐PD‐1 antibody treatment. Data represent the means ± SEM. Unpaired two‐tailed Student's *t*‐test was used for comparisons (B). n.s., no significance. See also Figure S2.

Given the malignant nature of AYP tumors, we evaluated their susceptibility to immune checkpoint blockade. To this end, we treated AYP mice with anti‐PD‐1 antibodies for three weeks (Figure 2A). Stomach weights remained comparable between treated and control groups (Figure 2B). Neither the incidence nor the morphology of metastatic lesions was altered by anti‐PD‐1 treatment (Figures 2C and S2A). Histopathological analysis confirmed the absence of therapeutic response: gastric tissues from both groups displayed mixed‐type adenocarcinoma with extensive immune infiltration (Figures 2D and S2B). Immune profiling revealed only a modest reduction of neutrophil and regulatory T cells (Treg) and group 3 innate lymphoid cells (ILC3) frequencies, with no substantial changes in other immune subsets (Figure S2C). Together, these results demonstrate that AYP tumors are intrinsically resistant to anti‐PD‐1 immunotherapy, despite a densely infiltrated tumor microenvironment (TME).

### 2.3 scRNA‐Seq Analysis of AYP Tumor Cells and Immune Microenvironment

**FIGURE 3:**
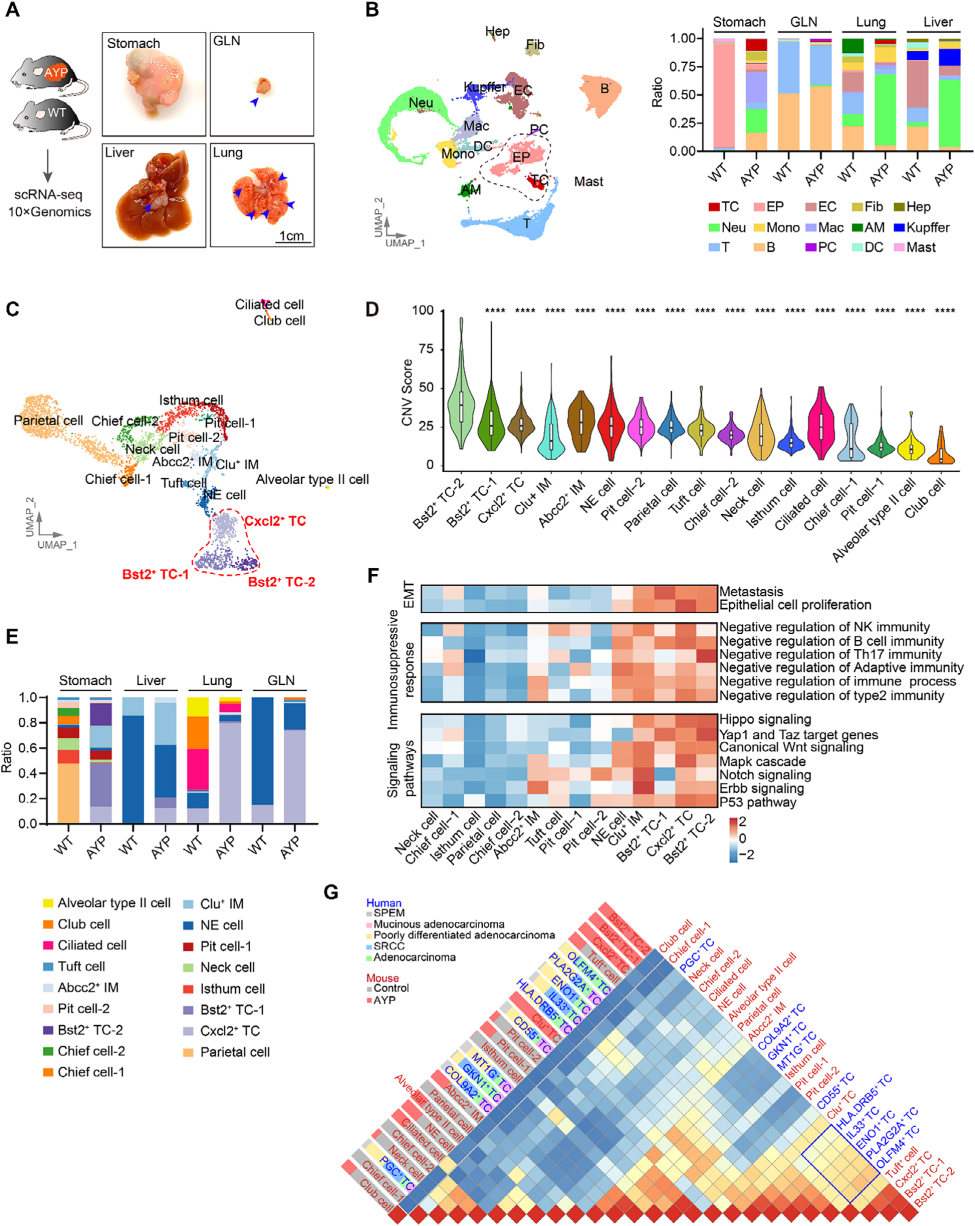
scRNA‐seq analysis of tumor cells from AYP GC mice. (A) The stomach (excluding forestomach), GLN and visible metastatic lesions in the liver and lung were collected from AYP mice (4 m after tamoxifen injection) with WT counterparts as controls, and then subjected to scRNA‐seq with 10× Genomics platform. Scale bar, 1 cm. (B) UMAP of cell subclusters derived from the indicated organs of control WT mice and AYP mice. colored by inferred different cell subclusters. Relative proportion of these cell subclusters in the indicated organs of AYP and WT control mice, Hep, hepatocyte; AM, alveolar macrophages; Mast, mast cell; PC, plasma cell; TC, tumor cell; DC, dendritic cell; Fib, fibroblast; Mono, monocyte; EP, epithelial cell; EC, endothelial cell; Mac, macrophage; Neu, neutrophil. (C) UMAP of gastric epithelial cells derived from WT and AYP mice, colored by inferred epithelial cell subclusters. NE, neuroendocrine cell; TC, tumor cell. (D) Copy number variation (CNV) analysis of epithelial subclusters. (E) Fraction of gastric epithelial cells derived from WT and AYP mice, colored by inferred epithelial cell subclusters. (F) Gene set variation analysis (GSVA) of representative gene sets associated with the shown pathways among epithelial cell subclusters. (G) Heatmap depicted the Pearson's correlations between epithelial cell subclusters from human (blue) and mouse (red) GC samples. The relative proportion of each epithelial cell subclusters in human or mouse GC tissues is indicated upper left side of the heatmap, colored by mouse GC model or human GC subtypes. Data represent the means ± SEM. One‐way ANOVA with Dunnett's post hoc analysis was used for comparisons (D). ^****^, *p* < 0.0001. See also Figure S3.

To dissect the mechanism driving tumorigenesis and resistance to immunotherapy of AYP tumors, we performed single‐cell RNA sequencing (scRNA‐seq) of stomach (excluding forestomach), GLN and visible metastatic lesions in liver and lung from AYP mice, and counterparts of WT mice (Figure 3A). After quality control and normalization [27, 28], 49,845 high‐quality single cells were annotated into 15 major cell lineages (Figure 3B) [29, 30, 31]. Three tumor cell clusters (Bst2^+^ TC‐1, Bst2^+^ TC‐2 and Cxcl2^+^ TC) were identified in AYP GC mice based on somatic copy number variations (CNVs) analysis among gastric epithelial cells (Figure 3C–E). In addition, gene set variation analysis (GSVA) revealed that these AYP tumor cells displayed significant enrichment of Hippo signaling and Yap/Taz target gene signatures, along with features of epithelial–mesenchymal transition (EMT) and immunosuppression (Figure 3F). To assess clinical relevance, we compared AYP tumor cell transcriptomes with an unpublished scRNA‑seq dataset from 26 gastric cancer patients using Garnett [29, 32, 33]. AYP tumor cells closely correlated with human poorly differentiated gastric adenocarcinoma (including signet‐ring cell carcinoma), with high correlation coefficients for signature genes such as HLA‐DRB5 (r = 0.4505, *p* < 0.0001), IL33 (r = 0.5389, *p* < 0.0001), ENO1 (r = 0.6317, *p* < 0.0001), PLA2G2A (r = 0.6599, *p* < 0.0001) and OLFM4 (r = 0.6955, *p* < 0.0001) (Figure 3G).

A prominent common feature across primary and metastatic sites (except GLN) was pronounced neutrophil infiltration (Figures 3B and S3A–C). GSVA analysis showed that Cebpe^+^, CD55^+^, Cd300ld^+^ and Tlr2^+^ neutrophil subsets displayed characteristics of polymorphonuclear myeloid‐derived suppressor cells (PMN‐MDSCs) (Figure S3D), key mediators of systemic immunosuppression clinically associated with poor immunotherapy responses. Additionally, Fos^+^ and Cd274^+^ neutrophils exhibited strong immunosuppressive signatures relative to Top2a^+^ and Ccl3^+^ neutrophils (Figure S3D). Consistent with this transcriptomic profile, flow cytometry confirmed accumulation of immunosuppressive SiglecF^+^ neutrophils at gastric tumor as well as lung and liver metastatic lesions post GC induction (Figure S3E). Moreover, depletion of neutrophils in AYP mice substantially inhibited gastric tumor development and metastasis (Figure S3F–H), suggesting the essential role of neutrophils in AYP GC progression.

### 2.4 BST2 Is a YAP‐Driven Signature Gene Upregulated in Anti‑PD‑1‑Resistant GC

**FIGURE 4:**
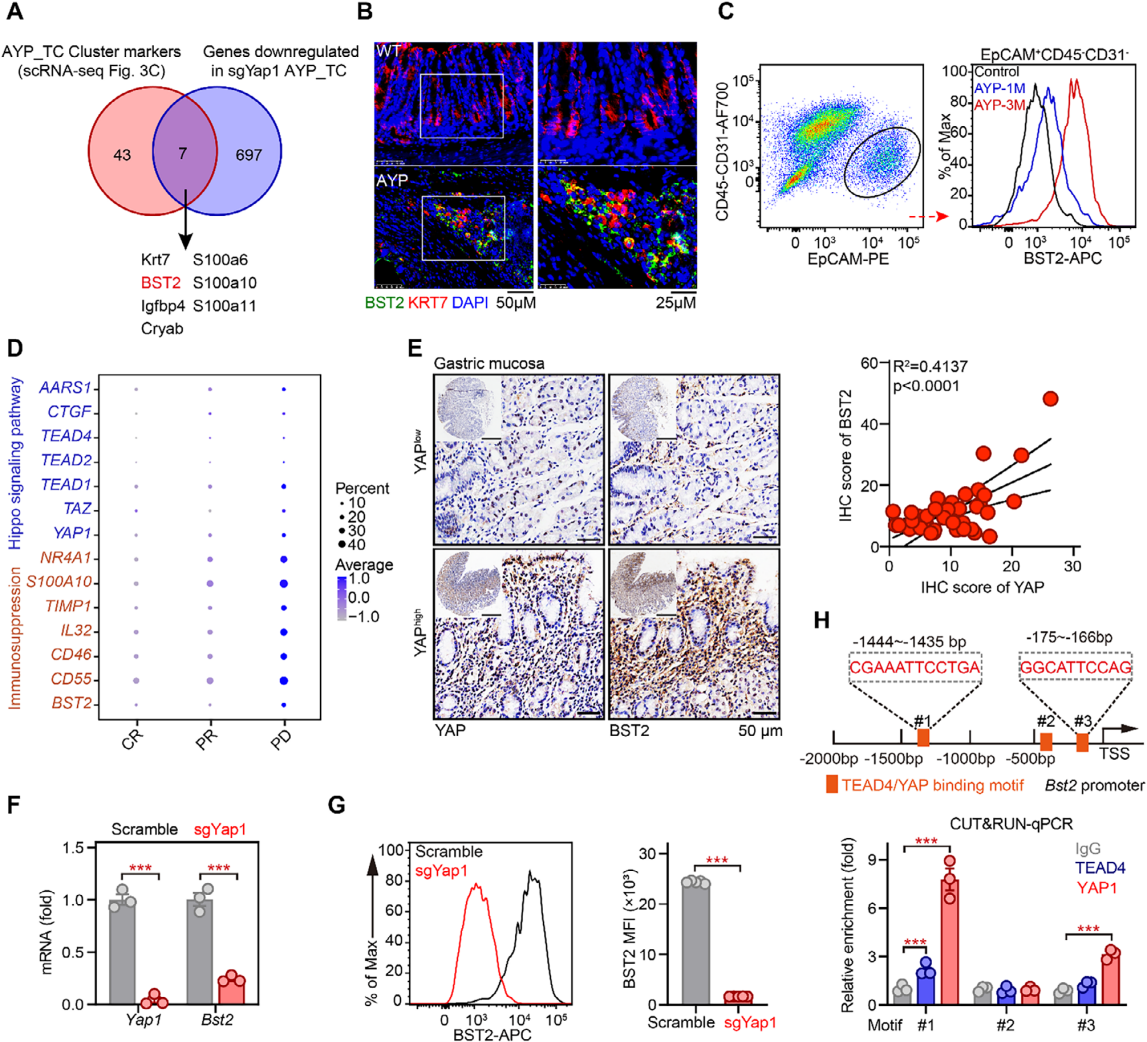
High expression of BST2 driven by YAP1 associates with anti‐PD‐1 resistance in GC. (A) Venn diagram showed the overlapping hits of signature genes of AYP tumor cells (scRNA‐seq) and down‐regulated genes after YAP1 knockout in AYP tumors (Bulk RNA‐seq). (B) Expression of BST2 and KRT7 of gastric sections from WT and AYP GC mice were determined by immunofluorescent staining. DAPI dye was used to stain cell nucleus. (C) Flow cytometry analysis of BST2 expression of gastric epithelial cells (EpCAM^+^CD45^−^CD31^−^) from WT and AYP GC mice. (D) Bubble plot showing the expression levels of genes associated with hippo signaling pathway (AARS1, CTGF, TEAD4, TEAD1, TAZ and YAP1) and immunosuppression (NR4A1, S100A10, TIMP1, IL32, CD46, CD55 and BST2) in gastric tumor tissues from GC patients with progression disease (PD), partial response (PR) or complete response (CR) to anti‐PD‐1 therapy. (E) Immunohistochemistry (IHC) staining of YAP and BST2 in sections of human gastric samples. Each point represents one human sample. (F and G) AYP tumor cells were infected with lentiviral particles harboring sgRNA targeting *Yap1*. The mRNA levels of *Yap1* and *Bst2* were determined by RT‐qPCR (F, *n* = 4 biological replicates per group), and the surface protein level of BST2 was analyzed by flow cytometry (G, *n* = 5 biological replicates per group), mean fluorescence intensity (MFI) of BST2 was calculated. (H) CUT&RUN‐qPCR analysis of the binding capacity of YAP1 and TEAD4 to predicted binding motif in the promoter of Bst2 (*n* = 3 biological replicates per group). Data represent the means ± SEM. Pearson correlation analysis was used for comparisons (E). Unpaired two‐tailed Student's *t*‐test was used for comparisons (F, G). Two‐tailed two‐way ANOVA with Sidak's multiple comparisons was used for comparisons (H). ^***^, *p* < 0.001. See also Figure S4.

To elucidate the mechanisms by which AYP tumor cells orchestrate an immunosuppressive and neutrophil‐rich tumor microenvironment, we first confirmed YAP hyperactivation in AYP tumor cells by assessing the levels of YAP expression and *Ctgf* and *Cyr61* expression (Figure S4A–D), and the effect of pharmacological TEAD4 inhibition [34] (Figure S4E–G). Integrated analysis of AYP tumor cell signature genes and down‐regulated genes after YAP1 knockout identified BST2 as a top candidate (Figures 3C and 4A; Figure S4H). Immunofluorescence demonstrated co‑expression of BST2 and KRT7 specifically within tumor tissues, with no detectable BST2 in normal gastric epithelium (Figure 4B). In addition, flow cytometry revealed increased BST2 expression of gastric epithelial cells along GC progression (Figure 4C). Clinically, BST2 and YAP1 transcription was markedly higher in anti‐PD‐1 non‑responders (progressive disease, PD) than in responders (complete/partial response, CR/PR) (Figure 4D), mirroring the expression patterns of established immunosuppressive markers such as *NR4A1* and CD55 [35, 36, 37, 38].

We next explored the potential link between YAP and BST2 expression in human gastric tissues. Indeed, YAP immunohistochemical (IHC) scores positively correlated with BST2 IHC scores across clinical specimens (Figure 4E), and co‐immunostaining revealed YAP and BST2 co‑expression in AYP tumor cells (Figure S4I), indicative of a direct link between YAP activation and the upregulation of BST2. To directly test whether YAP1 drives BST2 expression, we generated YAP1‐knockout AYP tumor cells using CRISPR/Cas9. Loss of YAP1 resulted in a significant reduction of both BST2 mRNA and surface protein levels (Figure 4F,G). Moreover, CUT&RUN‐qPCR further demonstrated direct binding of the YAP1‐TEAD4 complex to the *Bst2* promoter (Figure 4H), establishing BST2 as a direct transcriptional target of YAP1. Together, these data identify BST2 as a YAP‑driven signature gene of AYP tumor cells and a clinically relevant biomarker of anti‐PD‐1 resistance in GC.

### 2.5 BST2 Promotes Immunosuppression by Engaging Its Receptor PIRA2 on Neutrophils

**FIGURE 5:**
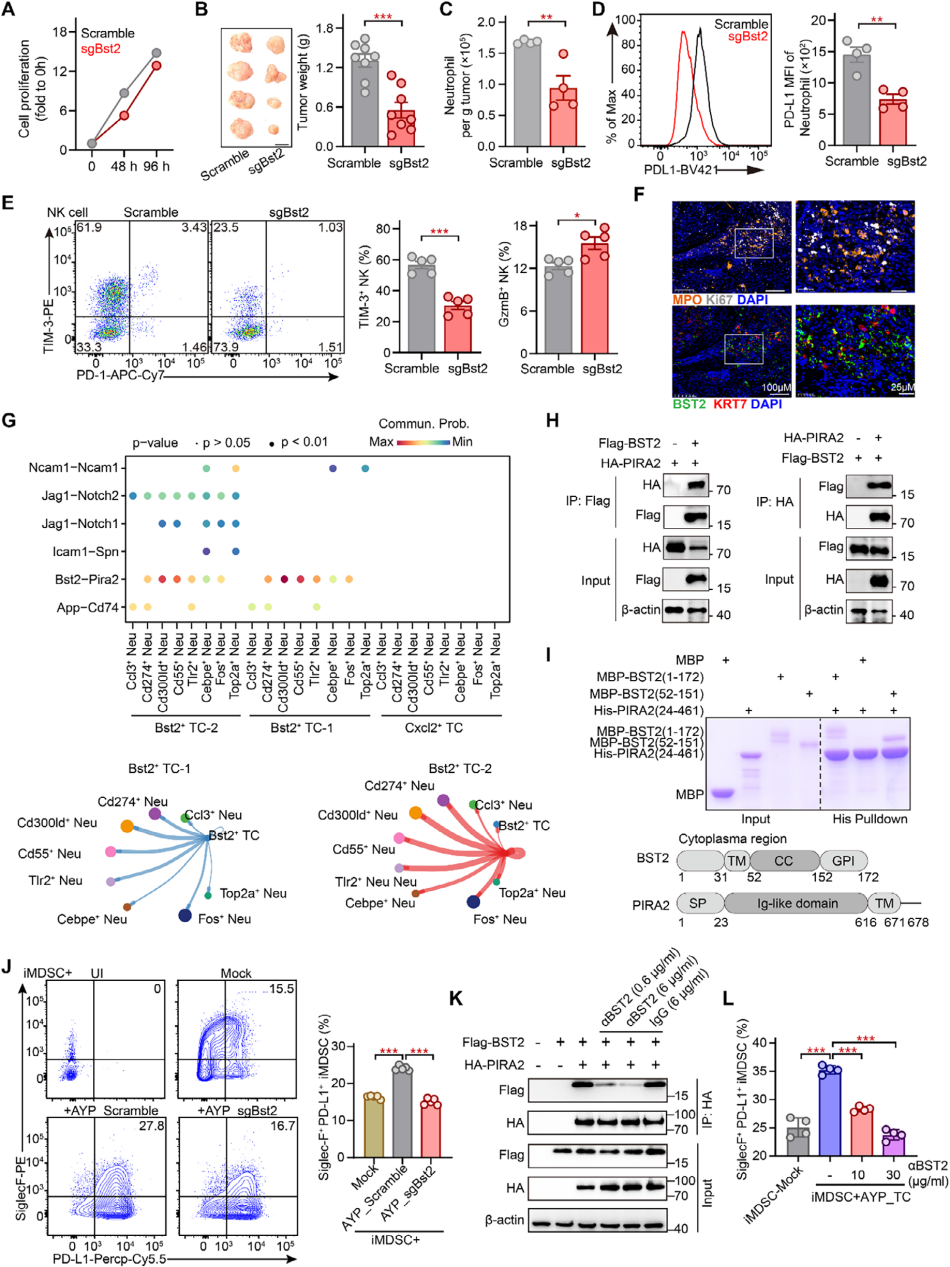
BST2 regulates immune escape of AYP tumor cells by reprogramming neutrophils. (A) Cell proliferation of BST2‐KO and control AYP tumor cells (*n* = 6 biological replicates per group). (B) Tumor growth in C57BL/6 WT mice after they were subcutaneously inoculated with AYP tumor cells and tumor weights were measured (*n* = 8 mice per group). Scale bar, 1 cm. (C) Flow cytometry analysis of neutrophils in the AYP tumor microenvironment. Cell numbers of tumor‐infiltrated neutrophils were normalized by tumor weights (*n* = 4 mice per group). (D) Flow cytometry analysis of PD‐L1 expression of AYP tumor infiltrated neutrophils (*n* = 4 mice per group). (E) TIM‐3, PD‐1, and GzmB expression of NK cells in the AYP tumor microenvironment were analyzed by flow cytometry (*n* = 5 mice per group). (F) Expression of MPO, Ki67, BST2, and KRT7 in gastric tissues from AYP mice were determined by immunofluorescent staining. DAPI dye was used to stain the cell nucleus. (G) Bubble plots exhibited significant ligand‐receptors pairs that contribute to signaling sending from Bst2^+^ TC and Cxcl12^+^ TC to the indicated neutrophil subclusters. The dot color and size represent the calculated communication probability and p‐values, computed from a one‐sided permutation test. Netvisual circle plot showed the interaction networks between Bst2^+^ TC and neutrophil subclusters. (H) Co‐IP assay showing the exogenous interaction of Flag‐tagged BST2 with HA‐tagged PIRA2 in HEK293FT cells. Cell lysates were incubated with Flag or HA beads overnight, and resulting immunoprecipitates were then subjected to immunoblotting analyses using anti‐Flag or anti‐HA antibodies. β‐actin was used as internal control. (I) Schematic diagram of domains of BST2 and PIRA2, and the direct interaction of Bst2‐PIRA2 was detected by his‐pulldown assay. The indicated proteins were visualized with Coomassie blue staining (CBB). (J) Flow cytometry analysis of siglecF and PD‐L1 expression on iMDSC co‐cultured with either scramble or sgBst2 AYP tumor cells (*n* = 6 biological replicates per group). (K) Co‐IP assay showing the exogenous interaction of Flag‐tagged BST2 with HA‐tagged PIRA2 in the presence of anti‐BST2 antibodies. (L) Flow cytometry analysis of SiglecF and PD‐L1 expression on iMDSC cocultured with AYP tumor cells in the presence of anti‐BST2 antibody (*n* = 4 biological replicates per group). Data represent the means ± SEM. Unpaired two‐tailed Student's *t*‐test was used for comparisons (B–E). One‐way ANOVA with Dunnett's multiple comparisons test was used for comparisons (J and L). ^*^, *p* < 0.05; ^**^, *p* < 0.01; ^***^, *p* < 0.001. See also Figure S5.

We hypothesized that BST2 contributes to immune evasion of AYP tumor cells. To test this, we generated BST2‐knockout AYP tumor cells (Figure S5A). While BST2 deletion showed marginal influence on cell proliferation in vitro (Figure 5A), it significantly impaired in vivo tumor formation and growth of AYP tumor cells (Figures 5B and S5B). Importantly, BST2 depletion led to a marked reduction of cell number and PD‐L1 expression of tumor infiltrated neutrophils (Figure 5C,D), accompanied by decreased exhaustion level of natural killer (NK) and CD4^+^ T cells, and increased production of Granzyme B (GzmB) in NK and CD4^+^ T cells (Figure s5E and S5C), underscoring the indispensable role of BST2 in mediating immune suppression of AYP tumor cells.

Immunostaining revealed close spatial association of BST2^+^ tumor cells and MPO^+^ neutrophils in AYP gastric tumor tissues (Figure 5F), implying direct cell interaction of BST2^+^ tumor cells and neutrophils. Consistently, ligand‐receptor interaction analysis revealed strong crosstalk between BST2^+^ tumor cells (BST2^+^ TC‐1/2) and immunosuppressive neutrophil subsets (Cd274^+^, CD55^+^, Cd300ld^+^, Tlr2^+^) via Bst2‐Pira2 ligand‐receptor pairs (Figures 5G and S5D). Given that BST2 is a known ligand for human leucocyte immunoglobulin‐like receptor A4 (LILRA4, also known as ILT7) [39, 40], and that PIRA2 [41], the murine ortholog of human LILR, is highly expressed on neutrophil (Figure S5E), we hypothesized that BST2 interacts with PIRA2 on neutrophils. Co‐immunoprecipitation confirmed BST2‐PIRA2 interaction (Figures 5H and S5F). Truncation and pulldown assays further delineated the molecular interface: the coiled‐coil domain of BST2 directly binds to the Ig‐like domain of PIRA2 via two distinct interfaces (Figures 5I and S5G).

To functionally assess the BST2‐PIRA2 axis, we co‐cultured bone marrow cells with AYP tumor cells under MDSC‐differentiating conditions [42]. AYP tumor cells significantly increased the proportion of immunosuppressive neutrophils co‑expressing SiglecF and PD‑L1, an effect that was markedly attenuated by BST2 knockout (Figure 5J), or by BST2‐blocking antibody (Figures 5K,L and S5H). Collectively, these results identify BST2 as an immune checkpoint required for AYP tumor growth in vivo, and demonstrate that AYP tumor cells educate neutrophils via BST2‐PIRA2 signaling to drive immunosuppression.

### 2.6 BST2 Enables AYP Tumor Cells Liver Metastasis by Educating Kupffer Cells

**FIGURE 6:**
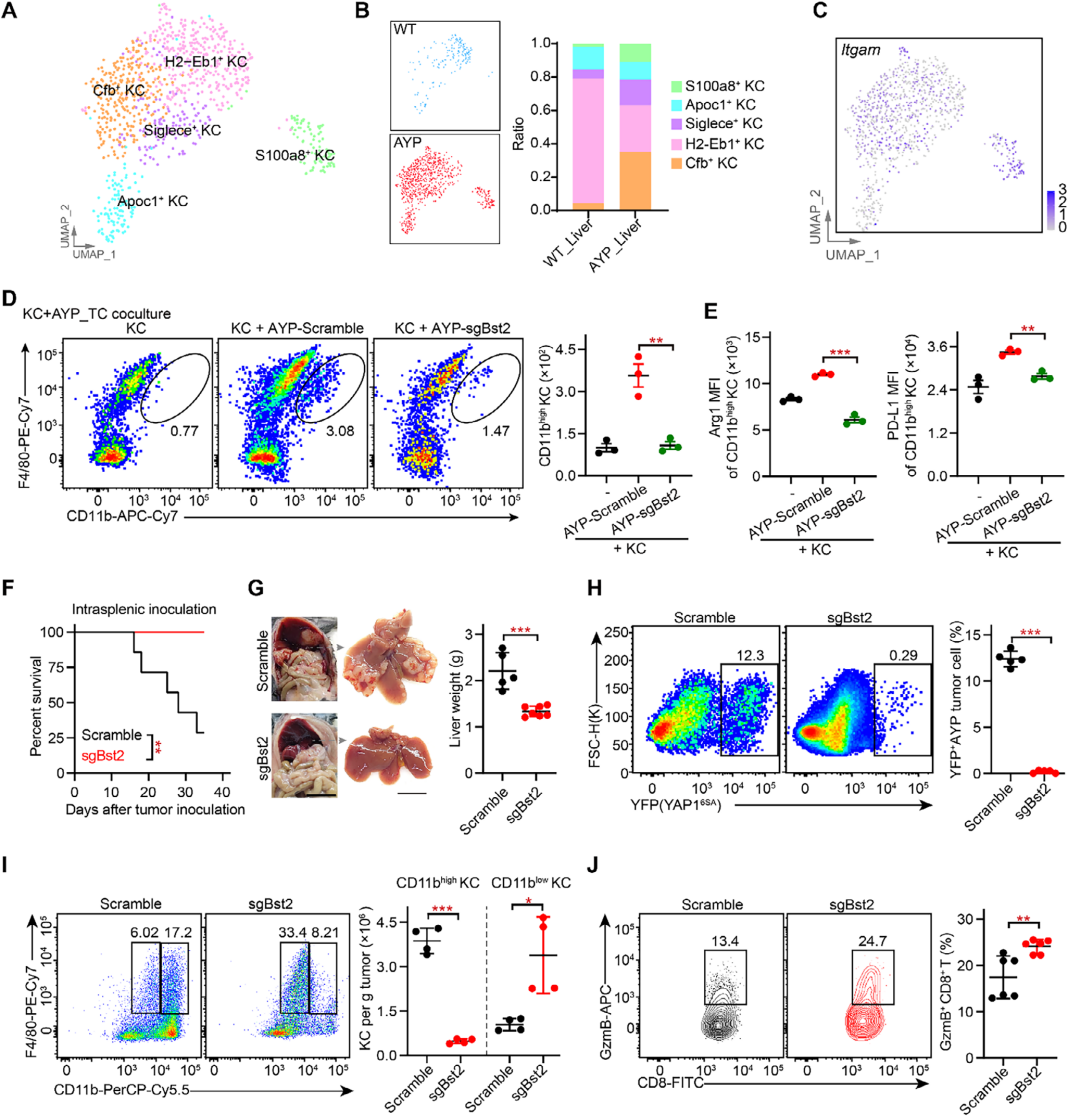
BST2 facilitates liver metastasis of AYP tumor cells. (A) UMAP of Kupffer cell (KC) subclusters derived from WT control and AYP mice, colored by inferred KC subclusters. (B) UMAP of KC subclusters, colored by different samples, and the relative proportion of inferred KC subclusters of WT and AYP mice were summarized. (C) UMAP showed the expression level of *Itgam* (coding CD11b) in different KC subclusters. (D) Liver resident KC defined as CD45^+^CD11b^low^F4/80^high^, were sorted by flow cytometry, and then co‐cultured with AYP tumor cells. The frequency of immunosuppressive CD11b^high^F4/80^high^ KC were determined by flow cytometry and cell number of CD11b^high^F4/80^high^ KC was calculated (*n* = 3 biological replicates per group). (E) Flow cytometry analysis Arg1 and PD‐L1 expression of CD11b^high^ KC cocultured with either AYP‐Scramble or AYP‐sgBst2 tumor cells. MFI of Arg1 and PD‐L1 was calculated (*n* = 3 biological replicates per group). (F) Survival of WT mice after intra‐splenic inoculation of AYP tumor cells (*n* = 7 mice per group). (G) Macroscopic picture showed the liver metastatic foci of the WT mice inoculated with AYP tumor cells via intra‐spleen injection, and liver weights were measured (*n* = 5 and 7 mice, respectively). Scale bar, 1 cm. (H) Flow cytometry analysis of liver metastatic AYP tumors cells (YFP‐YAP1^6A^) in WT recipient mice (*n* = 5 and 7 mice, respectively). (I) Flow cytometry analysis of liver CD11b^high^ and CD11b^low^ KC in WT recipient mice. KC number was normalized by tumor weights (*n* = 4 mice per group). (J) GzmB expression of liver CD8^+^ T cells in WT recipient mice was determined by flow cytometry analysis (*n* = 6 mice per group). Data represent the means ± SEM. Unpaired two‐tailed Student's *t*‐test was used for comparisons (D,E,H–J). Log‐rank (Mantel‐Cox) test was performed for survival comparison (F). ^*^, *p* < 0.05; ^**^, *p* < 0.01; ^***^, *p* < 0.001. See also Figure S6.

Given the high expression of PIRA2 on liver Kupffer cells (KC) (Figure S5E), we investigated whether BST2 similarly instructs KC to foster a metastatic niche in the liver. Transcriptional profiling of KC from AYP mice revealed accumulation of immunosuppressive KC subsets (S100a8^+^, Siglece^+^, Cfb^+^) and a concomitant reduction in immunostimulatory H2‐Eb1^+^ KC (Figures 6A,B and S6A). Moreover, GSVA analysis confirmed the immunosuppressive signatures of S100a8^+^ KC subsets, with relative high expression of *Itgam* (encoding CD11b) (Figures 6C and S6B).

In co‐culture assay, AYP tumor cells significantly increased the number of immunosuppressive CD11b^high^ Kupffer cells [43] (Figure 6D), and upregulated the immunosuppressive markers Arg1 and PD‑L1 (Figures 6E and S6C); these effects were abrogated upon BST2 knockout (Figures 6D,E and S6C). In addition, by using a model of liver metastasis by splenic injection of AYP tumor cells, we found that BST2 deficiency significantly prolonged host survival (Figure 6F). Consistently, BST2 loss profoundly reduced hepatic metastatic tumor burden (Figure 6G), and AYP tumor cells frequency (Figure 6H). Importantly, BST2 deletion led to a significant reduction of CD11b^high^ KC (Figure 6I). While T cells number were stable within liver metastatic lesions, GzmB production in effector CD8^+^ T cells was significantly increased after BST2 knockout (Figures 6J and S6D), indicative of enhanced anti‑tumor immunity. Together, these results demonstrate that AYP tumor cells employ BST2 to educate Kupffer cells, thereby promoting an immunosuppressive niche that facilitates liver metastasis.

### 2.7 Dual Blockade of BST2 and PD‐1 Suppress Primary GC and Eradicate Liver Metastases

To further establish the clinical relevance of BST2 as a driver of immunotherapy resistance, we evaluated BST2 expression in clinical specimens from GC patients treated with anti‐PD‐1 therapy. Notably, BST2 was substantially upregulated in tumors from patients with progressive disease (resistant) compared to those achieving complete response (sensitive) (Figure S7A,B). This association extended across multiple cancer types, reinforcing BST2 as a pan‐tumor marker of anti‐PD‐1 resistance (Figure S7A,B).

**FIGURE 7:**
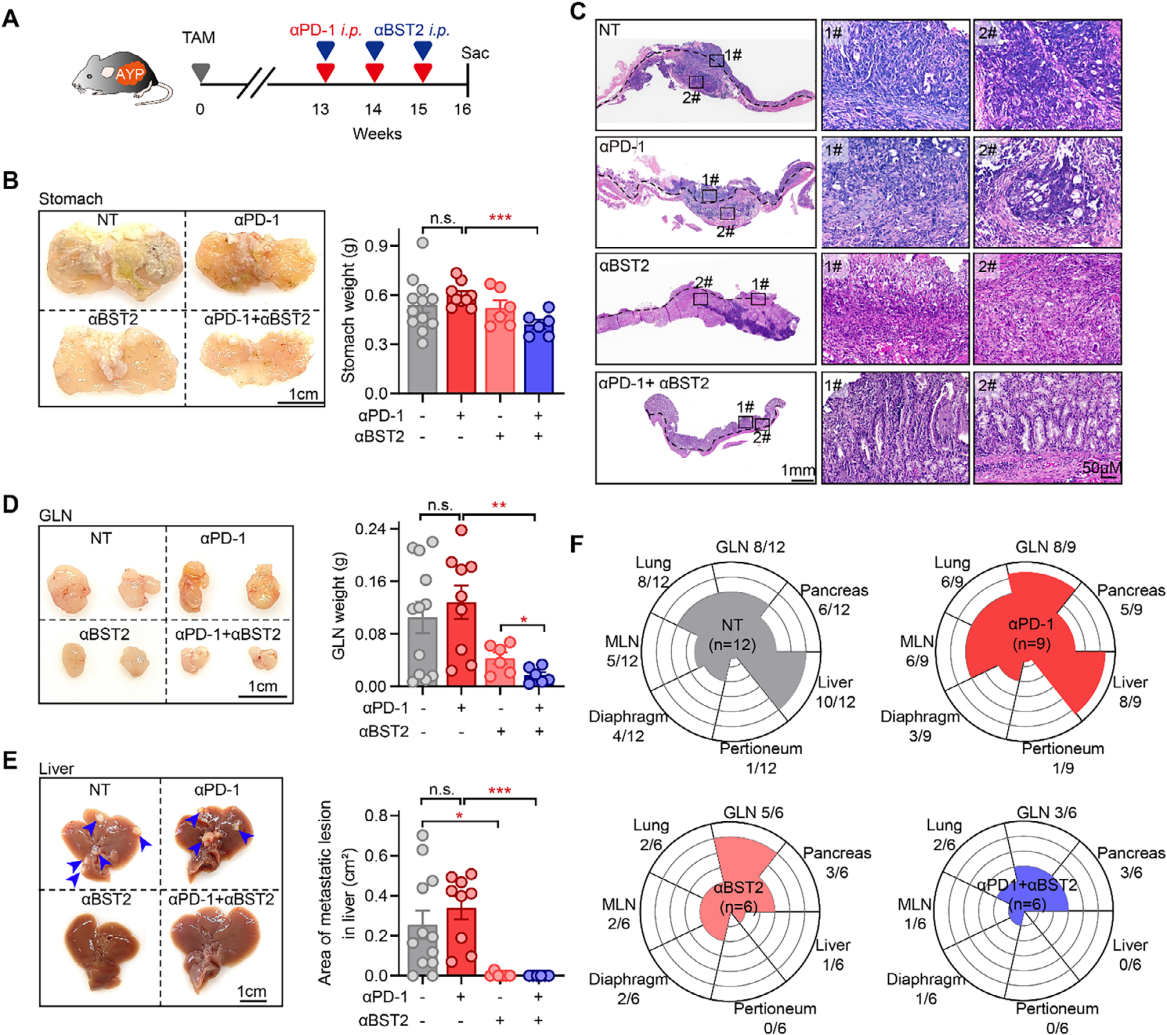
Synergistic blockade of BST2 and PD‐1 inhibits AYP tumorigenesis and metastasis. (A) Schematic diagram of anti‐PD‐1 and anti‐BST2 treatment in AYP mice. AYP mice (12 weeks after TAM injection) were intraperitoneally injected with anti‐PD‐1 antibodies (3 mg/kg) and/or anti‐BST2 antibodies (200 µg/mouse) weekly for 3 weeks, and were sacrificed to examine gastric pathological and immune features at 1 week post the last anti‐PD‐1 and anti‐BST2 antibodies injection. (B) Macroscopic picture showed the gastric tissues opened along the greater curvature from anti‐PD‐1 and/or anti‐BST2 antibodies treated mice, the stomach weights ware measured (*n* = 12, 9, 6, 6 mice, respectively). Scale bar, 1 cm. NT indicated not treated. (C) Representative pictures showed the H&E staining of gastric sections from AYP mice treated with anti‐PD‐1 and/or anti‐BST2 antibodies. (D) Macroscopic picture showed metastatic lesions of GLN of AYP mice with synergistic blockade of PD‐1 and BST2, and the bar graph showed weights of GLN (*n* = 12, 9, 6, 6 mice, respectively). Scale bar, 1 cm. (E) Macroscopic picture showed visible metastatic lesions (blue arrow) of liver of AYP mice treated with anti‐PD‐1 and/or anti‐BST2 antibodies. The area of liver metastatic lesions in AYP mice was measured with ImageJ based on macroscopic images and summarized in the bar graph (*n* = 12, 9, 6, 6 mice, respectively). Scale bar, 1 cm. (F) Petal graphs showed metastatic incidence of anti‐PD‐1 and/or anti‐BST2 antibodies treated AYP mice (*n* = 12, 9, 6, 6 mice, respectively). Each radius indicated the metastatic frequencies of the indicated organs, the outermost ring corresponded to 100%. Data represent the means ± SEM. One‐way ANOVA with Dunnett's multiple comparisons test was used for comparisons (B,D,E). ^*^, *p* < 0.05; ^**^, *p* < 0.01; ^***^, *p* < 0.001; n.s., no significance. See also Figure S7.

We next assessed whether BST2 blockade could sensitize AYP tumors to anti‐PD‐1 therapy (Figure 7A). Combination treatment with anti‑BST2 and anti‐PD‐1 antibodies reduced stomach weights by 29.7% compared to anti‐PD‐1 monotherapy (Figure 7B) and induced substantial histopathological restoration, characterized by reconstituted glands and differentiated epithelial structures (Figure 7C). Strikingly, BST2 monotherapy alone markedly reduced hepatic and GLN metastases (Figure 7D–F); its combination with anti‐PD‐1 completely eradicated visible hepatic metastases in all animals and achieved an additional suppression of GLN metastasis than single agents (Figure 7D–F).

Collectively, these results demonstrate that targeting BST2 sensitizes intrinsically resistant GC to anti‐PD‐1 immunotherapy, suppressing primary tumor growth but fully regressing established liver metastases.

## 3 Discussion

### 3.1 A Faithful Preclinical Model Unveils the Pathogenic Synergy of YAP Hyperactivation and TP53 Loss in Driving Refractory GC

GCs harboring concurrent YAP hyperactivation and TP53 loss represent an aggressive, metastasis‐prone subsets with intrinsic resistance to immunotherapy, yet a mechanistic understanding of this co‐occurrence has remained elusive. In this study, we established a GEMM (AYP) that, for the first time, faithfully recapitulates the cardinal features of human refractory GC: profound histopathological heterogeneity (including diffuse, mixed, and signet‐ring subtypes), multi‐organ metastasis, and innate resistance to anti‐PD‐1 therapy despite abundant immune infiltration [44]. Single cell transcriptomic profiling confirmed that AYP tumor cells closely align with poorly differentiated human GC subtypes, underscoring the model's translational relevance.

Beyond modelling, our work delineates the distinct and synergistic pathological roles of YAP hyperactivation and TP53 loss. Notably, YAP hyperactivation alone induced only hyperplasia, while TP53 loss alone failed to initiate gastric tumorigenesis [45] and primarily drove premalignant evolution [46, 47]. In sharp contrast, their “double‐hit” combination precipitated a qualitative leap in malignant progression, producing fully penetrant, metastatic adenocarcinomas. These findings establish that the aggressive phenotype of this GC subset is not merely additive but synergistic, arising from the cooperative interaction between two core oncogenic and tumor‐suppressive pathways. In addition, several studies have established that sustained Notch activation [17, 48], or combined loss of E‑cadherin and Tp53 [16, 49], in parietal cells precursors induce their dedifferentiation into multipotential progenitor‑like cells, ultimately leading to adenoma formation. Our AYP model is consistent with these findings, as YAP1^6A^‑YFP‑positive tumor cells originate from these Atp4b^+^ cells.

### 3.2 BST2 Is a YAP‐Driven Immune Checkpoint That Orchestrates Immunosuppression and Therapy Resistance

A central discovery of this study is the identification of BST2 as a bona fide immune checkpoint and a direct transcriptional target of YAP in tumor cells. This mechanistic link provides a molecular explanation for the refractory nature of YAP‐hyperactivated GCs. Of particular clinical relevance, we found that BST2 expression in human GC specimens correlates with poor response to anti‐PD‐1 therapy. BST2 is a type II transmembrane protein [50, 51], that engages ILT7 on plasmacytoid dendritic cells (pDCs) to suppress type I interferon responses [39]. It also promotes macrophage M2 polarization and interacts with PIRA2 on smooth muscle cells to drive immunosuppression [41, 52]. While BST2 has been sporadically implicated in cancer progression [41, 52, 53], our work defines its causal role in immunotherapy resistance and establishes its position within a druggable oncogenic signaling axis.

Mechanistically, we demonstrate that tumor cell‐derived BST2 engages its cognate receptor PIRA2 on neutrophils and liver‐resident Kupffer cells, instructing them toward an immunosuppressive phenotype. PIRA2 is the murine orthologue of human LILRs, expressed on myeloid cells and B cells [54]. It associates with FcRγ (containing immunoreceptor tyrosine‐based activation motifs, ITAMs) to drive proinflammatory signaling [55], and CD1d has recently been identified as its functional ligand in macrophage activation in nonalcoholic fatty liver disease [56]. The BST2‐PIRA2 ligand‐receptor interaction represents a previously unrecognized mode of tumor–neutrophil/Kupffer cell crosstalk that fosters a permissive niche for both primary tumor growth and metastatic seeding. Indeed, in the liver metastasis model, CD8^+^ T cells showed increased Granzyme B production after BST2 knockout, whereas BST2 depletion enhanced Granzyme B production in nature killer cell and CD4^+^ T cells, but not CD8^+^ T cells in the primary gastric tumor tissues. Moreover, given that PIRA2 is broadly expressed on neutrophils, Kupffer cells, macrophages, and dendritic cells, we propose that GC cells employ the BST2–PIRA2 axis in a context‐dependent manner, educating distinct environmental cell subsets at primary and metastatic sites to enforce immune evasion and therapy resistance throughout disease progression.

### 3.3 Clinical and Therapeutic Implications of Targeting the YAP‐BST2 Axis

The robust correlation between BST2 expression and resistance to anti‐PD‐1 therapy across multiple cancer types including GC positions BST2 not only as a biomarker of immunotherapy non‐response but also as a therapeutically actionable target. This clinical relevance is particularly striking given the paradox of “cold” therapeutic responses in “hot” immune‐infiltrated GCs [29, 57, 58]: despite the presence of dense immune infiltrates, objective response rates to anti‐PD‐1 monotherapy remain below 15% [59]. Our findings resolve this paradox by identifying BST2 as a dominant immune checkpoint that overrides PD‐1 blockade, rendering the tumor ecosystem refractory to T cell reinvigoration.

Importantly, we provide preclinical proof‐of‐concept that dual blockade of BST2 and PD‐1 not only sensitizes intrinsically resistant tumors to immunotherapy but also eradicates established liver metastases—a feat rarely achieved refractory GC models. In addition, the reduction in LN metastasis by anti‑BST2 therapy, despite low BST2^+^ tumor cells in LN, maybe attributed to BST2 expression on non‑tumor cells. Of note, pDCs express high levels of BST2 and are enriched in metastatic lymph nodes, where they promote Treg cells and establish an immunosuppressive microenvironment [60, 61, 62, 63]. Anti‑BST2 antibody may therefore reduce LN metastasis by targeting pDCs, consistent with previous study showing that BST2 blockade depletes pDCs and attenuates T cell exhaustion in LN, thereby inhibiting tumor progression [64]. This combinatorial strategy thus represents a promising, mechanistically rationalized approach to overcome resistance in a high‐unmet‐need patient population. Given the pan‐cancer expression of BST2 and its association with ICB resistance, the therapeutic principle established here may extend beyond GC to other BST2‐high, immunotherapy‐refractory solid tumors.

Furthermore, the association between bacterial colonization and YAP‐BST2 expression opens new avenues for investigation. Bacterial infection activate YAP signaling in host cells via direct infection or the release of secreted factors. For instance, Helicobacter pylori (*H. pylori*) deliver the virulent protein CagA into gastric epithelial cells through its type IV secretion system (T4SS), leading to YAP activation [65]. Likewise, outer membrane vesicles (OMVs) released by Porphyromonas gingivalis (*P. gingivalis*) facilitate YAP nuclear translocation [66]. In our experimental condition, *S. anginosus* colonizes the superficial mucosa, while YAP upregulation mainly localizes to base glands, suggesting indirect activation via secreted factors. Whether persistent BST2 upregulation is driven by ongoing microbial stimuli or represents a stable YAP‐imprinted epigenetic state remains to be determined. Future studies leveraging longitudinal patient sampling and antibiotic intervention in preclinical models will be necessary to establish causality and explore the potential of combining BST2 blockade with microbiome modulation.

### 3.4 Limitations and Future Directions

Several questions remain to be addressed. First, the precise molecular events downstream of BST2‐PIRA2 engagement that license immunosuppressive reprogramming in neutrophils and Kupffer cells have not been fully elucidated. A high‐resolution structural analysis of the BST2‐PIRA2 complex would not only illuminate the mechanistic basis of this signaling axis but also facilitate the development of next‐generation blockade strategy with improved affinity and specificity. Second, while our study demonstrates therapeutic efficacy in a spontaneous, fully immunocompetent model, humanized antibodies to BST2 or small molecule antagonists are now warranted and evaluated their therapeutic efficacy in AYP model and humanized patient‐derived xenografts (PDXs) model to explore the translational potential of anti‐BST2 therapy. Finally, the interplay between BST2 and other established immune checkpoints (e.g., LAG3, TIGIT) in shaping the resistant tumor microenvironment remains an open area for future investigation.

## 4 Conclusion

In summary, this work establishes that YAP hyperactivation and TP53 loss synergize to drive aggressive, immunotherapy‐resistant gastric cancer via transcriptional induction of the BST2 immune checkpoint. By dissecting the BST2‐PIRA2 axis as a critical mediator of tumor, neutrophil/Kupffer cell crosstalk, we uncover a targetable vulnerability in refractory GC and provide a strong rationale for clinical evaluation of BST2/PD‐1 combinatorial blockade. More broadly, our findings illustrate how mechanistic interrogation of high‐fidelity preclinical models can illuminate both fundamental cancer biology and new therapeutic strategies for diseases with intractable clinical challenges.

## 5 Methods

### 5.1 Animals

*Atp4b^Cre‐ERT2^* (*Atp4b‐2A‐CreERT2*, C57BL/6 background) mouse was generated by homologous recombination in embryonic stem cells targeting the *Atp4b‐2A‐CreERT2* cassette to the stop codon of *Atp4b* by Shanghai Model Organisms Center (SMOC)*. Yap1^6A/+^* (H11‐LSL‐*Yap1^6A^*, C57BL/6 background) transgenic mouse was generated by inserting the CAG‐loxP‐Stop‐loxP‐*Yap1(6A, S46A/T68A/S94A/S112A/S149A/S382A)*‐YFP‐WPRE‐polyA cassette into the H11 locus on mouse chromosome 11 by SMOC. *Tp53^Flox/+^* mice were generously provided by Dr. Hongbin Ji (Westlake University, Hangzhou, China) [67]. All mice noted above were crossed with the C57BL/6 background. Genotyping PCR primers were shown in Table S1. For gastric tumorigenesis induction, adult mice at the age of 8–10 weeks were intraperitoneally administrated with 100 mg/kg tamoxifen (Sigma, T5648) dissolved in corn oil (ABCONE, C25834) for 3 consecutive days.

All mice were housed under specific pathogen‐free conditions in automated watered and ventilated cages on a 12 h light/dark cycle and handled in accordance with were the guidelines of the Institutional Animal Care and Use Committee of the Fudan University.

### 5.2 Cell Culture

MFC cells were obtained from the National Infrastructure of Cell Line Resource (Beijing, China), HEK293FT cells were obtained from Biofeng (Hunan, China), these cell lines were maintained in DMEM medium (Invitrogen). YTN16 cells were kindly provided by Dr. Tetsuya Tsukamoto (Fujita Health University, Toyoake, Japan) [68] and cultured in DMEM medium (Invitrogen) in the presence MITO^+^ serum extender (Corning) on type I collagen precoated dishes. All cell culture mediums were supplemented with 10% heat‐inactivated FBS (Biological Industries), and 1% penicillin‐streptomycin (Gibco) at 37°C with 5% CO_2_ in a humidified incubator (Thermo Scientific). Cells were passaged for ≤ 3 months from the frozen early‐passage stocks that had been received from the indicated sources. During the study, all cell cultures were periodically tested for mycoplasma using MycoAlert Mycoplasma Detection Kits (Lonza).

### 5.3 Primary Tumor Cell Culture Derived from Spontaneous AYP Mice

To obtain the primary tumor cell culture from primary GC foci of AYP mice. Gastric tumors from AYP mice were dissected into <1 mm pieces with sterile scissors and digested in RPMI 1640 medium containing 1 mg/mL Collagenase II (Sigma) and 100 µg/mL DNase I (Sigma) at 37°C in a water bath for 15 min. The cells suspensions were filtered with a 100 µm cell strainer (Corning Falcon). After a 5 min centrifugation at 1000 rpm, cell pellets were washed two times with complete RPMI 1640 medium supplemented with 10% FBS and 1% penicillin‐streptomycin. The cells were then resuspended in complete RPMI 1640 medium and incubated for 30 min in plastic dishes to promote the adhesion of fibroblasts. Cell culture media containing floating cell clusters were collected and transferred to new plastic dishes to allow the adhesion and expansion of AYP cells. These primary tumor cells were maintained in completed RPMI 1640 medium at 37°C with 5% CO_2_ in a humidified incubator (Thermo Scientific). Cells were passaged for ≤ 3 months from the frozen early‐passage stocks. The tumorigenicity and metastatic capacity of AYP cells were evaluated after orthotopic and subcutaneous inoculation in wild‐type mice with a C57BL/6 background.

### 5.4 Generation of Yap1 or Bst2‐Knockout AYP Cells

Yap1 or Bst2‐knockdown AYP cells used in this study were generated using the CRISPR‐Cas9‐based gene targeting approach. Briefly, sequences of single guide RNA (sgRNA) oligos targeting *Yap1* or *Bst2* genes were obtained from the GeCKO library (https://www.genscript.com/CRISPR‐gRNA‐library.html). The sequences of the sgRNA targeting *Yap1* were 5′‐CACCG ATACCCTTACCTGTCGCGAG‐3′, 5′‐CACCGCCAAGGCTGGACCCTCGTTT‐3′ and 5′‐CACCG GCCCAAGTCCCACTCGCGAC‐3′. The sequences of the sgRNA targeting *Bst2* were 5′‐CACCG CGCTCGCAACCCGTCTCTAC‐3′, 5′‐ CACCGTAGAGACGGGTTGCGAGCGC‐3′ and 5′‐CACCG GTGACACTTTGAGCACCAGT‐3′. Lentiviral particles harboring the indicated sgRNA targeting *Yap1* or *Bst2* genes were generated by Oligobio (Beijing, China), lentiviral particles containing scrambled sgRNA was used as control. AYP cells were infected with the lentiviral particles (MOI = 5) in the presence of polybrene (5 µg/mL, Sigma–Aldrich) at 37°C for 48 h. Puromycin (10 µg/mL, Sigma–Aldrich) was used to select AYP cells infected by the lentiviruses. Knockdown efficiency *Yap1* or *Bst2* was validated by RT‐qPCR and/or flow cytometry.

### 5.5 Co‐culture of iMDSC, Kupffer Cell With AYP Tumor Cells

To generate MDSCs from murine bone marrow (iMDSC), bone marrow cells were isolated from wild‐type mice and cultured in RPMI‐1640 medium supplemented with 10% heat‐inactivated FBS, 1% penicillin‐streptomycinm, GM‐CSF (40 ng/mL) and IL‐6 (40 ng/mL). The iMDSC were co‐cultured with AYP‐scramble or AYP‐sgBst2 tumor cells, respectively, at a ratio of 10:1 for 3 days. The expression of PD‐L1 and SiglecF iMDSC were determined by flow cytometry. Liver resident Kupffer cell (KC) defined as CD45^+^CD11b^low^F4/80^high^, were sorted by flow cytometry, and then co‐cultured with AYP tumor cells. Frequency of CD11b^high^F4/80^high^ KC and expression of PD‐L1 and Arg1 were analyzed by flow cytometry.

### 5.6 Liver Metastasis Model

Wild‐type mice were anesthetized with avertin. A small incision was made on the left flank using surgical scissors, and the spleen was carefully exteriorized. AYP tumor cells (1×10^6^ cells in 60 µL PBS) were then slowly injected into the spleen. Following the injection, the wound was sutured carefully. After recovery from anesthesia, the mice were returned to the housing cages.

### 5.7 Targeting Treatment of Spontaneous AYP Mice

AYP mice were first induced gastric tumorigenesis by tamoxifen (TAM) administration intraperitoneally (100 mg/kg) intraperitoneally. After GC induction by TAM injection. AYP mice were intraperitoneally treated with GLUP (1 mg/kg) or anti‐Ly6G antibodies (200 µg/mouse, Biolegend, Cat# 127649) twice per week, and anti‐PD‐1 antibodies (3 mg/kg, Hengrui) and/or anti‐BST2 antibodies (200 µg/mouse, Biolegend, Cat# 127031) once weekly for 3 weeks.

### 5.8 H&E Staining and Histological Analysis

Tumor tissues were fixed overnight in 4% paraformaldehyde at 4°C, embedded in paraffin, and cut into 3.5‐µm sections. Sections were stained with hematoxylin & eosin (H&E). Images were captured with Pannoramic DESK (3DHISTECH, Budapest, Hungary) and analyzed with Slideviewer (3DHISTECH, Budapest, Hungary). Images of stained primary GC and metastatic tissue sections were reviewed and scored by two pathologists in a double‐blind manner.

### 5.9 Immunohistochemical and Immunofluorescent Staining

Immunohistochemical and immunofluorescent staining was performed following standard protocols [23]. The antibodies used included those against human YAP (ab205270, Abcam,1:50), BST2 (222103, Zen‐bioscience, 1:50), mouse Bst2 (127030, Biolegend, 1:100), Krt7 (ab181598, Abcam, 1:100), and Myeloperoxidase (ab208670, Abcam, 1:100). Images were captured with Pannoramic DESK (3DHISTECH, Budapest, Hungary) and analyzed with Slideviewer (3DHISTECH, Budapest, Hungary).

### 5.10 Murine Tissue Preparation and Flow Cytometry

Stomach (excluding forestomach), whole gastric and mediastinal lymph node (GLN and MLN), and visible metastatic foci of liver, pancreas, peritoneum, diaphragm and lung of AYP mice were collected and dissected into <1 mm pieces with scissors and then digested in RPMI 1640 medium with 1 mg/mL Collagenase II (Sigma) and 100 µg/mL DNase I (Sigma) at 37°C for 30 min. The above cells suspensions were filtered with a 70 µm cell strainer (Corning Falcon). After a 5 min centrifugation at 400 g, the cells were resuspended with red blood cell lysis buffer (RBL, TIANGEN) and incubation 5 min at room temperature to deplete red blood cells. Peripheral blood was collected into EDTA containing tubes and incubated with RBL to deplete red blood cells. Subsequently, the cells were washed 2 times and kept in FACS buffer (1 × PBS containing 2% FBS and 2 mm EDTA) on ice before use. For the analysis of surface marker, single cell suspensions were first blocked with purified CD16/CD32 antibody (Biolegend) and then incubated with fluorophore‐conjugated anti‐mouse antibodies against CD45, CD11b, Ly6C, Ly6G. F4/80, CD11c, MHC‐II, PD‐L1, CD127, TCRβ, TCRγδ, CD3ε, CD4, CD8α, NK1.1, CD19, FAS, GL7, CD31, EpCAM, TIM‐3, PD‐1 and Bst2 in FACS buffer for 30 min at 4°C. For intracellular cytokine and transcription factors staining, the cells were fixed and permeabilized according to the users’ guide (Invitrogen) for further intracellular GzmB, IFN‐γ, Ki67, Foxp3, IL‐10, GATA3 and RORγt staining. Flow cytometry acquisition was performed on a 4‐laser BD LSRFortessaTM (BD) using FACSDiva software. The cells were gated based on the FSC‐A and SSC‐A followed by doublets exclusion with FSC‐H and FSC‐W. Dead cells were excluded with DAPI (Sigma) or LIVE/DEAD yellow (Invitrogen). Data was subsequently analyzed with FlowJo software (Tree Star). Antibodies used in this study were shown in Table S2.

### 5.11 Human Specimen Collection

Treatment‐naïve patients who received neoadjuvant anti‑PD‑1 antibody combined with platinum‑based doublet chemotherapy and subsequently underwent successful surgical resection were included in this study. Patients who did not undergo surgery after neoadjuvant therapy were excluded. Tumor resection was performed 4–12 weeks after the last dose. Tumor dynamics were monitored by MRI/PET‑CT at baseline and before surgery. Patients were classified according to the Tumor Regression Grade (TRG) as sensitive (TRG = 0, *n* = 45) or resistant (TRG = 3, *n* = 48). For BST2 IHC scoring, the integrated optical density (IOD) was measured using Image Pro Plus 6.0 and defined as the BST2 IHC score.

### 5.12 Immunoblotting and Immunoprecipitation

For immunoblotting assays, cells were lysed with NETN buffer (20 mm Tris·HCl pH 8.0, 100 mm NaCl, 0.5% NP‐40, and 1 mm EDTA) supplemented with micrococcal nuclease (NEB) and cocktail (TargetMol) on ice for 30 min. Cell lysates were then boiled with 5 × SDS loading buffer for 10 min. The proteins were separated using SDS‐PAGE, transferred to PVDF membranes (Bio‐Rad), and further incubated with indicated antibodies. Antibodies used for immunoblotting included those against β‐actin (A2228, Sigma–Aldrich, 1:5000), HA (3724, Cell Signaling Technology, 1:1000), Flag (F3165, Sigma–Aldrich, 1:5000) and HRP‐conjugated secondary antibodies including goat anti‐rabbit (31460, Thermo, 1:5000), and goat anti‐mouse (31430, Thermo, 1:5000). Western blotting images were captured by using a Mini Chemiluminescent Imaging and Analysis System (Beijing Sage Creation Science Co, LTD). For co‐immunoprecipitation experiments, cells were lysed with the above‐mentioned NETN buffer for 30 min on ice, with the resulting material subjected to centrifugation at 12 000 rpm for 10 min at 4°C. Resulting supernatants were then transferred into a new Eppendorf tube and incubated with either HA beads (GenScript) overnight at 4°C with rotation (50 rpm). These beads were subsequently washed three times with NETN buffer and boiled with 1 × SDS loading buffer. The immunoprecipitate was then detected by following the above‐described immunoblotting assay procedure.

### 5.13 Pulldown Assay

The recombinant proteins (MBP‐tagged Bst2 and His‐tagged Pira2) were immobilized on amylose resin or glutathione agarose resin, respectively, and then incubated with various target proteins in binding buffer (20 mm HEPES, pH 7.5, 250 mm NaCl, 10 mm β‐mercaptoethanol) at 4°C for 2 h. After incubation, the resins were washed three times with binding buffer, followed by elution using the same buffer supplemented with 10 mm maltose monohydrate (for MBP‐fused proteins). The immobilized proteins were subsequently detected by either Coomassie Brilliant Blue staining.

### 5.14 ChIP‐qPCR Assay

To determine the binding motif of Yap1/Tead4 in the promoter of Bst2, chromatin immunoprecipitation coupled with quantitative PCR (ChIP‐qPCR) using anti‐Yap1 (ab250272, abcam) or anti‐Tead4 (sc‐101184, Santa Cruz Biotechnology) antibody was conducted in AYP cells, ∼20 000 cells were used to isolate nuclei. ChIP‐qPCR samples were prepared using Hyperactive pG‐MNase CUT&RUN Assay Kit (Vazyme) according to the manufacturer's protocol. In both experiments, Primers used in this study are listed in Table S3.

### 5.15 RNA Extraction, Reverse Transcription and Quantitative Real‐Time PCR

Total RNA was extracted from cells or homogenized tissue samples with RNA extraction reagent (Vazyme) according to the manufacturer's instructions. A mass of 1 µg of RNA was used to synthesize the first cDNA strand with HiScript II Q RT SuperMix (Vazyme). Quantitative real‐time PCR (RT‐qPCR) was performed with Hieff qPCR SYBR Green Master Mix (Yeasen) using an Applied Biosystems 7500 system (Thermo). Actin was used as the internal control. Primers used in this study are shown Table S3.

### 5.16 scRNA‐Seq and Data Processing

The stomach (excluding forestomach), GLN, and visible metastatic lesions in the liver and lung were collected from AYP mice (4 m after tamoxifen injection) with WT counterparts as controls, and then subjected to scRNA‐seq. Subsequent scRNA‐seq was conducted by Novel Bio (Shanghai). scRNA‐seq data of all samples were normalized by using the Seurat package (version 3.0) [28] in R (version 4.2.2). Cells that expressed fewer than 200 genes or more than 5000 genes were excluded, as were the cells whose mitochondria and red blood cell gene content were greater than 30% and 1% of the total UMI count, respectively. The total number of transcripts in every single cell was normalized to 10 000. Highly variable genes, namely genes with very different levels of expression in different cells, were detected according to average expression (between 0.1 to 8) and dispersion (above 1) of the genes, followed by data scaling (subtracting the average expression) and centering (dividing standard deviation). Those variable genes were considered to account for cell‐to‐cell differences and were further used for principal component analysis (PCA). The first 50 principal components (PCs) were applied for UMAP analysis according to the eigenvalues.

### 5.17 Identification of Cell Types

We extracted transcriptomic data of cells from the expression profiles of all the single cells. We annotated the cells based on known cell lineage‐specific marker genes as epithelial cells (*Epcam, Muc5ac, Pgc*), tumor cells (TC) (*Krt7, Cdkn2a, Ybx3*), fibroblasts (*Col1a1, Col1a2, Col3a1*), endothelial cells (*Pecam1, Cd34, Plvap*), T cells (*Cd3e, Cd3d, Cd4, Cd8a*), B cells (*Cd79b, Ms4a1, Pax5*), plasma cells (*Iglc2, Igkc*), neutrophils (*S100a8, S100a9, Csf3r*), macrophages (*C1qa, C1qc, Apoe*), monocytes (*Arg1, Vcan*), dendritic cells (*Itgax, Htr7*) and mast cells (*Kit, Ms4a2*) [29, 30, 31].

### 5.18 CNV Estimation

Cells defined as fibroblasts were used as a reference to identify somatic copy number variations (CNVs) with the R package inferCNV (v0.8.2) of the Trinity CTAT Project (https://github.com/broadinstitute/inferCNV). We scored each cell for the extent of the CNV signal, defined as the mean of squares of CNV values across the genome. Putative malignant cells were then defined as those with a CNV signal above 0.05 and CNV correlation above 0.5.

### 5.19 Gene Set Variation Analysis (GSVA)

The enriched pathway analysis was performed on the epithelial cells with the R package GSVA. The gene sets indicated were downloaded from the molecular signature database (https://www.gsea‐msigdb.org/gsea/msigdb/index.jsp). Briefly, the average expression of every gene in each epithelial cell subtypes was calculated using the function of “AverageExpression” in the R package Seurat. Those genes that were expressed in at least one cell were included. Then, we utilized the function of “gsva” to calculate the score of every interested gene set in each epithelial cell subtype and showed the results by heatmap.

### 5.20 Statistical Analysis

All quantitative data were presented as mean ± SEM unless specified otherwise and were analyzed with GraphPad Prism 8.0 statistical software. Unpaired two tailed Student's *t*‐test was used for comparing two quantitative variables. Two‐way ANOVA with Sidak's multiple comparisons test was performed for multiple comparisons. Survival analysis was performed using the log‐rank test. In figures, asterisks were used as follows: ^*^, *p* < 0.05; ^**^, *p* < 0.01; and ^***^, *p* < 0.001. *p* < 0.05 were considered statistically significant.

### 5.21 Ethics Approval Statement

Animal procedures were approved by the Institutional Animal Care and Use Committee of Fudan University (Approval ID: IDM2024009).

## Author Contributions

W.Z. performed AYP model construction and functional experiments. S.W. performed H&E staining and constructed liver metastasis model. M.W., R.Y. and Y.M. performed scRNA‐seq analysis. J.Y., L.T., W.Q. and Y.T. performed biochemical analysis. L.S. and S.C. performed co‐culture assay. H.Z. and M.Z. performed IHC (F) staining. Y.H., W.W. and L.A. discussed the data. W.Z., S.J. and Z.Z. wrote the manuscript. Y.M., S.J. and Z.Z. supervised the project.

## Conflicts of Interest

Z.Z., S.J., and W.Z. have filed a patent (No. 2025108521664) for the development of AYP cells in China.

## Supporting information




**Supporting File 1**: advs77708‐sup‐0001‐SuppMat.docx.

**Supporting File 2**: advs77708‐sup‐0002‐SuppMat.pdf.

## Data Availability

The sequencing data generated in this study have been deposited in the National Genomics Data Center (NGDC) under accession codes OMIX011104 (https://ngdc.cncb.ac.cn/omix/preview/ae81l8M7).
