# Supplementary material for: Targeting the YAP‐BST2 Axis Overcomes Intrinsic Anti‐PD‐1 Resistance in Metastatic Gastric Cancer
Source: Adv Sci (Weinh). 2026 Sep 21:e77708. Online ahead of print. doi: 10.1002/advs.77708 (PMC13591348; doi:10.1002/advs.77708)

Figure 5H

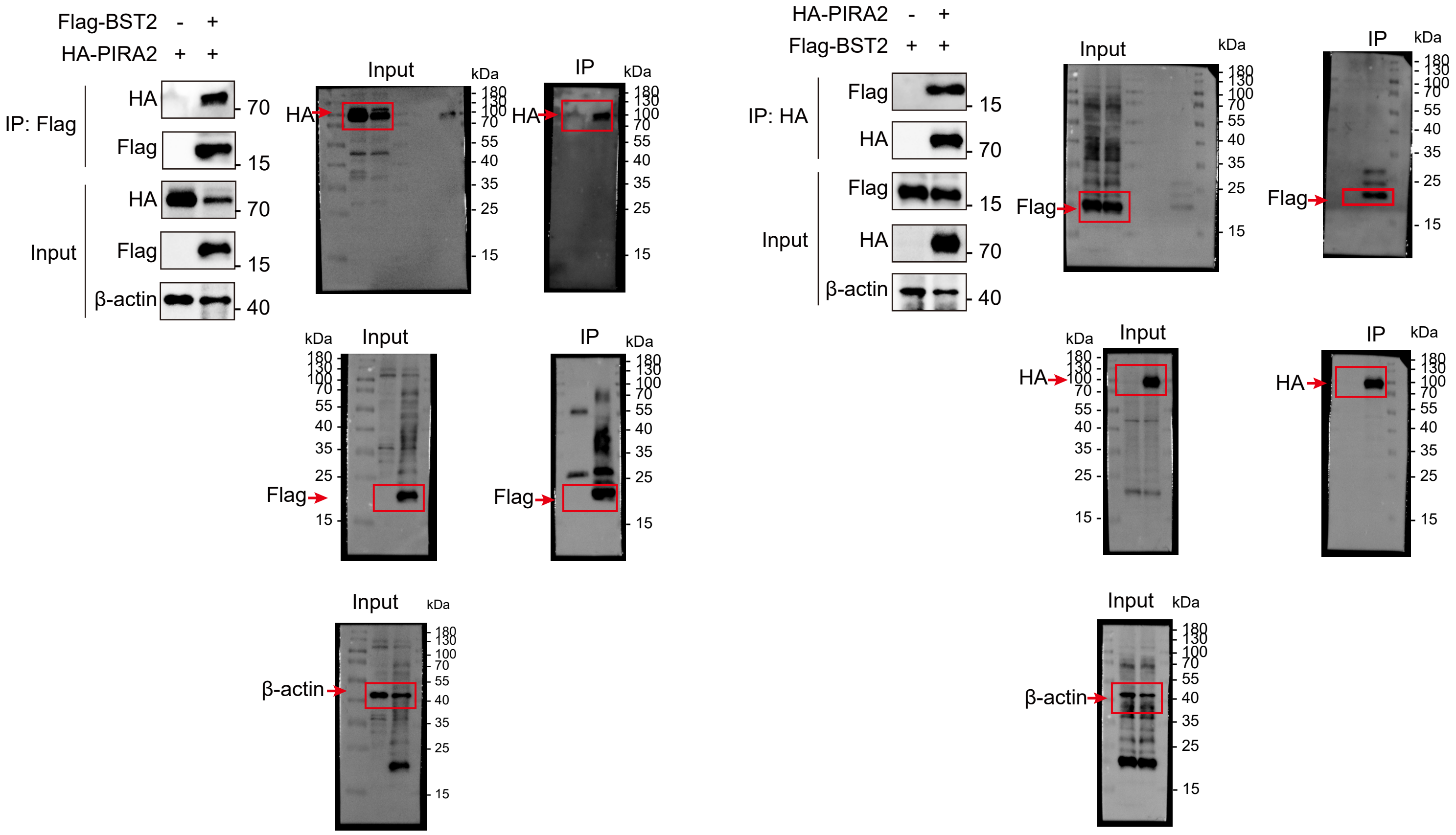

Figure 5I

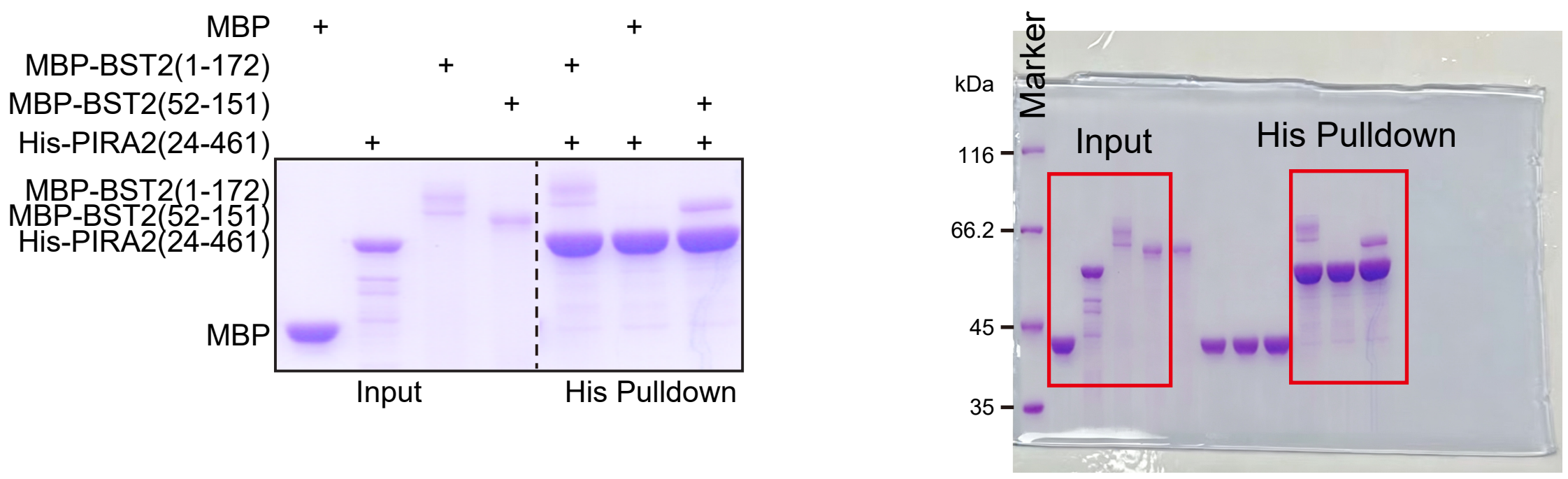

Figure 5K

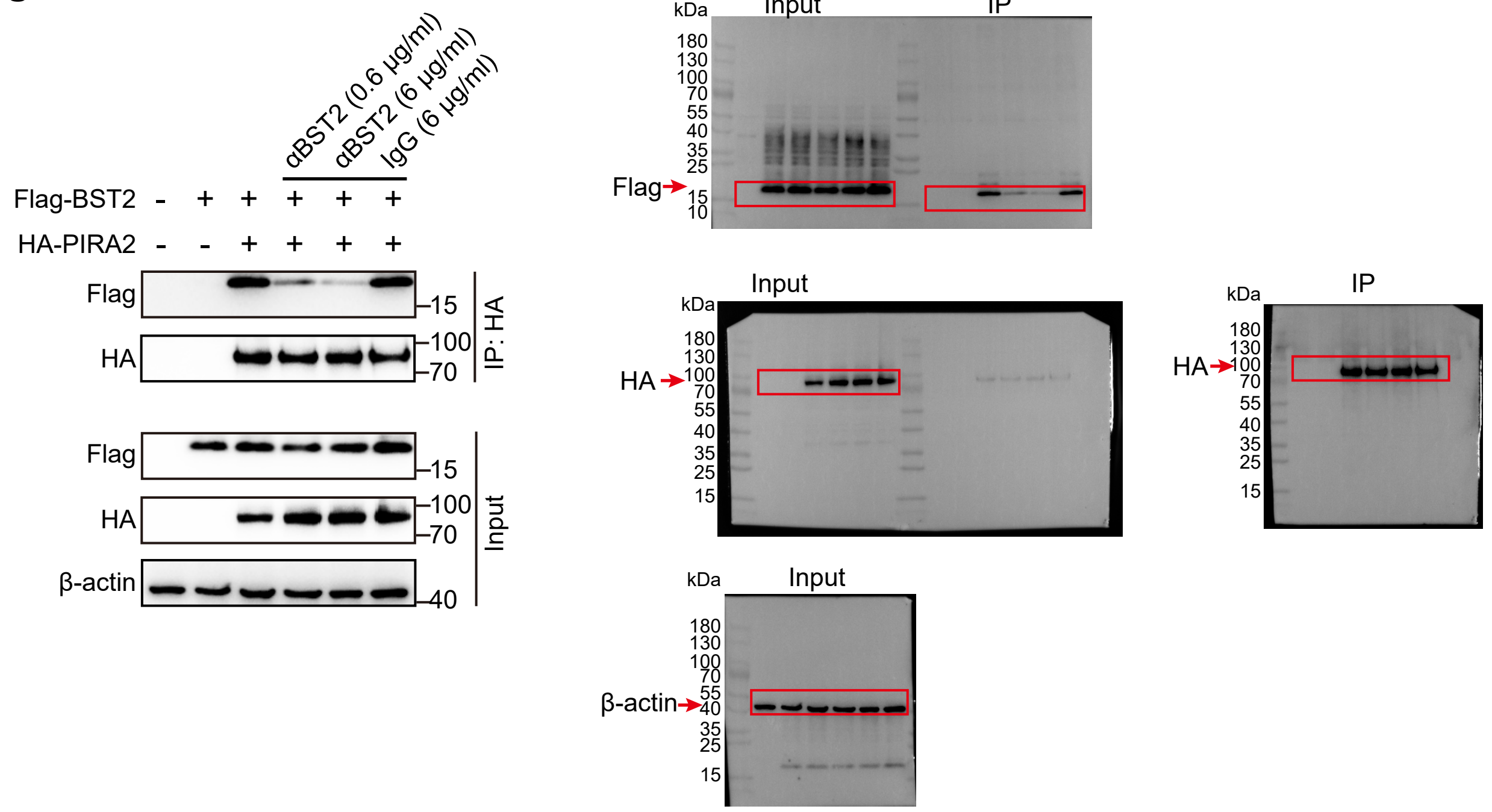

Figure S5F

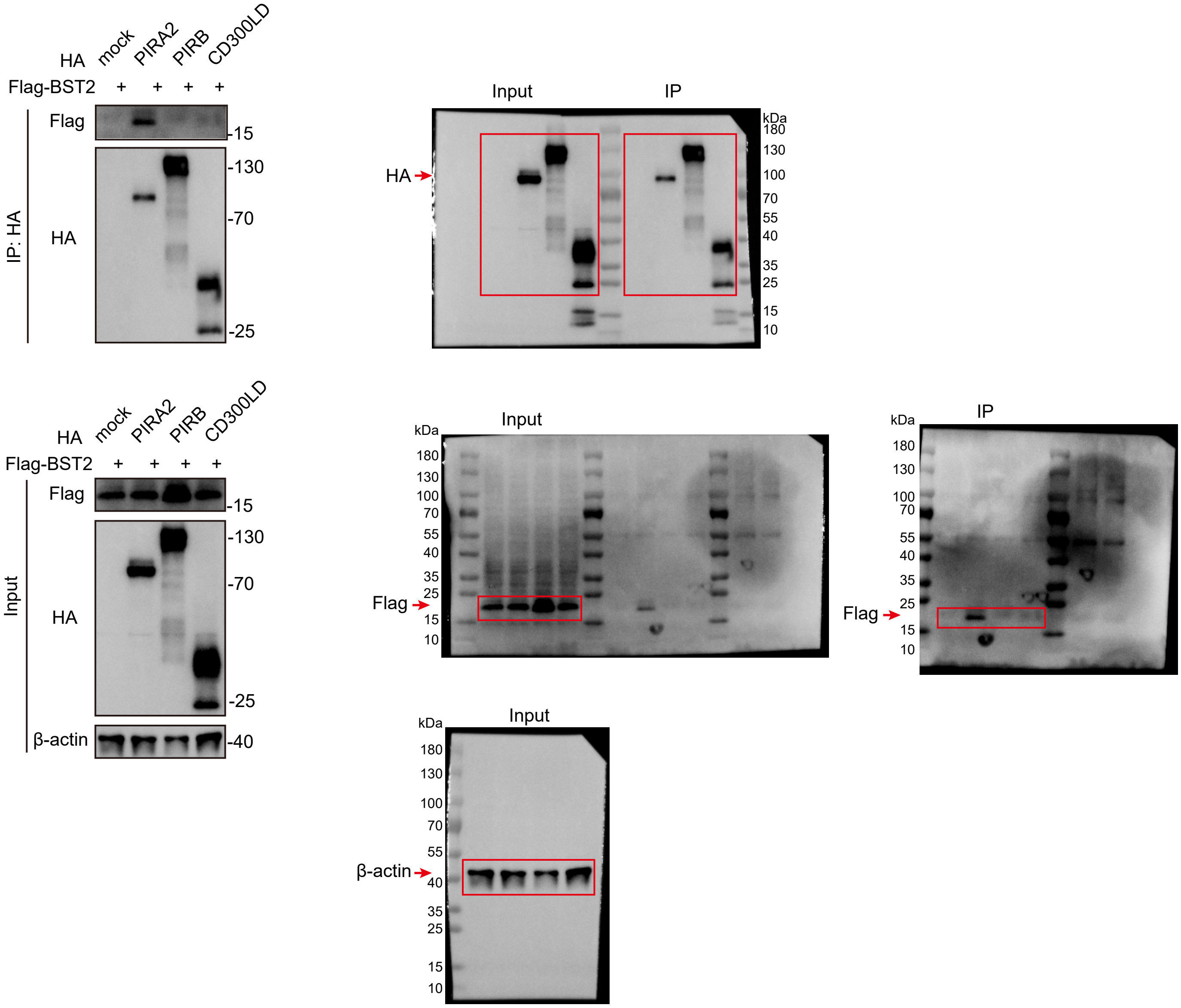

Supplement: Supplementary file 1 [file ADVS-9999-e77708-s001.pdf]
