# Supplementary material for: Targeting the YAP‐BST2 Axis Overcomes Intrinsic Anti‐PD‐1 Resistance in Metastatic Gastric Cancer
Source: Adv Sci (Weinh). 2026 Sep 21:e77708. Online ahead of print. doi: 10.1002/advs.77708 (PMC13591348; doi:10.1002/advs.77708)
Supplement: Supplementary file 2 [file ADVS-9999-e77708-s002.docx]

**Supplementary Information**

**Supplementary Figures (1~7) and Legends**

**Supplementary Tables (1-3)**

**Supplementary Figures (1~7) and legends**

**Figure S1. AYP mice develop metastatic GC**

(**A**) Schematic diagram of Hippo signaling pathway. In canonical Hippo signaling pathway, MST1/2 kinases initiate a phosphorylation cascade that activates LATS1/2, which in turn phosphorylate the transcriptional co-activators YAP and TAZ, sequestering them in the cytoplasm for subsequent degradation. MST4 kinase-mediated phosphorylation of YAP acts an additional mechanism of YAP inactivation. Upon dephosphorylation, YAP/TAZ translocate to the nucleus, where they bind transcription factors such as TEADs to activate transcription of downstream target genes.

(**B**) Genetic alteration of MST1, MST2, LATS2, YAP1, TAZ, TEAD1, TEAD2 and TEAD4 in human gastric cancer.

(**C**) RNAscope of *S. anginosus* 16S rRNA, and immunohistochemistry (IHC) staining of YAP of stomach after *S. anginosus* infection (n = 5 mice per group).

(**D**) Atp4b mRNA level in different tissues based on the public datasets in the human protein atlas. nTPM, normalized protein-coding transcripts per million.

(**E**) H&E staining of gastric sections from WT and AYP mice without (w/o) TAM injection.

(**F**) Representative H&E staining of gastric sections from AP and AY mice after TAM administration. AP (*Atp4b^Cre-ERT2^;Tp53^fl/fl^*, Tp53 loss alone) and AY (*Atp4b^Cre-ERT2^;LSL-Yap1^6A/6A^*, YAP activation alone).

(**G**) H&E staining showed GC metastasis to GLN, peritoneum, pancreas, liver, diaphragm, mediastinal lymph node and lung from AYP mice. The red bold T indicated the tumor area in the metastatic organs.

(**H**) Summary of histologic classification, tumor differentiation and inflammation score of metastatic lesions in AYP mice.

(**I** and **J**) Flow cytometry analysis of metastatic tumors cells (YFP-YAP1^6A^) in AYP mice (I) and frequency of YFP^+^ tumor cell was summarized in (J, n = 6 mice).

Related to **Figure 1**.

**Figure S2. AYP mice showed** **intrinsic resistance to anti-PD-1 therapy**

(**A**) Representative macroscopic picture showed metastatic lesions (bule arrow) of control and anti-PD-1 antibody treated AYP mice. Scale bar, 1 cm.

(**B**) GC primary location, histologic and Lauren’ classification, tumor differentiation state, invasive range, inflammation score and tumor regression grade (TRG) of the gastric lesions from control and anti-PD-1 antibody treated AYP mice were scored by pathologists.

(**C**) Flow cytometry analysis of gastric tumor-infiltrated immune cell subsets from AYP mice with or without anti-PD-1 antibody therapy (n= 4 and 8 mice, respectively). Representative flow chart showed the gating strategy of CD45^+^ immunocytes, CD4^+^ T, CD8^+^ T and neutrophil (Ly6C^low^ Ly6G^+^). ILC, innate lymphoid cell; CTL, cytotoxic T lymphocyte; Treg, regulatory T cell; Breg, regulatory B cell; GCB, germinal center B cell.

Data represent the means ± SEM. Two-tailed two-way ANOVA with Sidak's multiple comparisons was used for comparisons (**C**) *, p < 0.05. Related to **Figure 2.**

**Figure S3. Immunosuppressive neutrophils accumulate in AYP tumor microenvironment.**

(**A**) UMAP of neutrophil subclusters from AYP mice, colored by inferred neutrophil subclusters.

(**B**) Bubble plot showing the expression levels of signature genes for the eight neutrophil subclusters.

(**C**) UMAP of neutrophil subclusters, colored by different samples. Relative proportion of inferred neutrophil subclusters derived from primary GC and metastatic foci of AYP mice were summarized.

(**D**) GSVA of representative gene sets associated with shown pathways among neutrophil subclusters.

(**E**) Flow cytometry analysis of total (Ly6C^low^ Ly6G^+^) and SiglecF^+^ neutrophils in the indicated organs of AYP mice at 2 and 4 months (n = 7 or 12 mice, respectively) after GC induction by TAM injection.

(**F**) AYP mice (9 weeks after TAM injection) were intraperitoneally injected with anti-Ly6G antibody (200 μg) twice per week. Flow cytometry analysis of peripheral neutrophil of AYP mice treated with anti-Ly6G antibody (n = 4 mice per group).

(**G**) Macroscopic picture showed the gastric tissues from AYP mice after anti-Ly6G antibody treatment (n = 4 and 3 mice, respectively), the stomach weights were measured. Scale bar, 1 cm.

(**H**) Petal graph showed metastatic incidence of anti-Ly6G antibody treated AYP mice (n= 4 and 3 mice, respectively). Each radius indicated the metastatic frequencies of indicated organs, the outermost ring corresponds to 100%.

Data represent the means ± SEM. Unpaired two-tailed Student’s *t*-test was used for comparisons (**E-G**). *, p < 0.05; **, p < 0.01; ***, p < 0.001. Related to **Figure 3**.

**Figure S4. Expression of YAP1 in AYP tumor cell and targeting TEAD4 inhibit AYP GC progression**

(**A**) Representative picture showed the cell culture of primary AYP tumor cells derived from spontaneous AYP mice. Scale bar, 10 μm.

(**B**) mRNA level of *Yap1* in MFC and AYP tumor cells was determined by RT-qPCR (n = 3 biological replicates per group).

(**C**) YFP (Yap1^6A^) fluorescence intensity of MFC, YTN16 and AYP tumor cells was assessed by flow cytometry (n = 5 biological replicates per group).

(**D**) RT-qPCR analysis of YAP1 target genes (*Cyr61* and *Ctgf*) in MFC and AYP tumor cells (n = 3 biological replicates per group).

(**E**) Schematic diagram of GLUP design and GLUP treatment of AYP mice. AYP mice were intraperitoneally injected with GLUP (1mg/kg) twice per week.

(**F**) Macroscopic picture showed the gastric tissues from AYP mice w/wo GLUP treatment, the stomach weights were measured and summarized in the bar graph (n= 7 and 5 mice, respectively).

(**G**) Petal graph showed metastatic incidence of AYP mice w/wo GLUP treatment (n= 7 and 5 mice, respectively). Each radius indicated the metastatic frequencies of indicated organs, the outermost ring corresponds to 100%.

(**H**) Volcano showed the different expressed genes in AYP tumor cells after YAP1 knockout.

(**I**) Expression of Ki67, BST2 and YAP in AYP gastric sections were analyzed by immunofluorescent staining. DAPI dye was used to stain cell nucleus.

Data represent the means ± SEM. Unpaired two-tailed Student’s *t*-test was used for comparisons (**B-D** and **F**). *, p < 0.05; ***, p < 0.001. Related to **Figure 4**.

**Figure S5. BST2 regulates immune escape of AYP tumor cells *via* reprogramming neutrophils.**

(**A**) Flow cytometry analysis of surface BST2 level of BST2-KO and control AYP tumor cells.

(**B**) Tumor growth in C57BL/6 WT mice inoculated with AYP tumor cells. Scale bar, 1 cm.

(**C**) TIM-3, PD-1 and GzmB expression of CD4^+^ T or CD8^+^ T cells in the AYP tumor microenvironment were analyzed by flow cytometry (n = 5 mice per group).

(**D** and **E**) Bubble plot showing the expression levels of *Bst2* in GLN, liver, lung and stomach from WT and AYP GC mice (D), and the expression levels of *Pira2* in indicated cell clusters (E).

(**F**) Co-IP assay showing the exogenous interaction of Flag-tagged BST2 with HA-tagged PIRA2, PIRB and CD300LD in HEK293FT cells. Cell lysates were incubated with HA beads overnight, and resulting immunoprecipitates were then subjected to immunoblotting analyses using anti-Flag or anti-HA antibodies. β-actin was used as internal control.

(**G**) Structure of the BST2-PIRA2complex, showing the residues of BST2-PIRA2 mediating their interaction.

(**H**) Flow cytometry analysis of SiglecF and PD-L1 expression on iMDSC cocultured with AYP tumor cells anti-BST2 antibodies treatment.

Data represent the means ± SEM. Unpaired two-tailed Student’s *t*-test was used for comparisons (**A** and **C**). **, p < 0.01; ***, p < 0.001; n.s., no significance. Related to **Figure 5.**

**Figure S6. BST2 potentiates liver metastasis of AYP tumor cells**

(**A**) Bubble plot showing the expression levels of signature genes for the five KC subclusters.

(**B**) GSVA of representative gene sets associated with shown pathways among KC subclusters.

(**C**) Flow cytometry analysis Arg1 and PD-L1 expression of CD11b^high^ KC cocultured with AYP tumor cells.

(**D**) Flow cytometry analysis of liver infiltrated CD4^+^ T, CD8^+^ T, Treg and PD1^+^Tim3^+^CD8^+^ T (Tex) in WT mice with intra-splenic inoculation of AYP tumor cells (n = 6 mice per group).

Data represent the means ± SEM. Unpaired two-tailed Student’s *t*-test was used for comparisons (**D**). n.s., no significance. Related to **Figure 6**.

**Figure S7. Increased BST2 level in multiple anti-PD-1-resistant human tumors**

(**A**) Immunohistochemical staining of BST2 of stomach, lung, liver, esophagus, colon and breast tumors sections from human patients. STAD, stomach adenocarcinoma; NSCLC, non-small cell lung cancer; HCC, hepatocellular carcinoma; ESCA, esophageal carcinoma; COAD, colon adenocarcinoma; BRCA, breast cancer.

(**B**) BST2 expression of tumor tissues from STAD, NSCLC, HCC. ESCA. COAD and BRCA cancer patients. Each circle indicated one cancer patient and number (N) were marked below.

Data represent the means ± SEM. Two-tailed two-way ANOVA with Sidak's multiple comparisons was used for comparisons (**B**). *, p< 0.05; **, p< 0.01. ***, p< 0.001. Related to **Figure 7**.

**Supplementary Tables (1-3)**

**Table S1: list of primers for mouse genotyping**

| Mouse name | Primer 1 | Primer 2 | Primer 3 | Primer 4 |
| --- | --- | --- | --- | --- |
| *Atp4b^Cre-ERT2^* | GCCCCACTACAGCAACCCTC | TGTTAACCCCACAGCTCCGA | CCAGGACAGACCCACAGAGGA | CGGACCGACGATGAAGCAT |
| *LSL*-*Yap1^6A/6A^* | TTAATCCAGCCTGACTCCAACTA | CTGCCCTTACCTTACTACCACTGT | TCTCGAGATGAGGCCACAGA | CTTGTAGTTGCCGTCGTCCT |
| *Tp53^fl/f^* | CACAAAAACAGGTTAAACCCAG | AGCACATAGGAGGCAGAGAC |  |  |

**Table S2: list of key resources**

| Reagent or Resource | Source | Identifier/Dilution |
| --- | --- | --- |
| **FACS, IF, IHC and WB antibodies** | | |
| Alexa Fluor 700 anti-mouse CD45 (clone 30-F11) | Biolegend | Cat# 103128; RRID: AB_493715 |
| PerCP/Cyanine5.5 anti-mouse TCRβ (clone H57-597) | Biolegend | Cat# 109228; RRID: AB_1575173 |
| PE anti-mouse TCRγδ (clone UC7-13D5) | Biolegend | Cat# 107508; RRID: AB_345265 |
| FITC anti-mouse CD3ε (clone 145-2C11) | Biolegend | Cat# 100306; RRID: AB_312671 |
| PE-Cy7 anti-mouse NK1.1 (clone PK136) | Biolegend | Cat# 108714; RRID: AB_389363 |
| PerCP/Cyanine5.5 anti-mouse CD127 (clone A7R34) | Biolegend | Cat# 135022; RRID: AB_1937273 |
| PerCP/Cyanine5.5 anti-mouse CD4 (clone GK1.5) | Biolegend | Cat# 100434; RRID: AB_893324 |
| FITC anti-mouse CD8a (clone 53-6.7) | Thermo Fisher | Cat# 11-0081-82; RRID: AB_464915 |
| PE anti-mouse CD8a (clone 53-6.7) | Thermo Fisher | Cat# 12-0081-85; RRID: AB_465532 |
| Alexa Fluor 700 anti-mouse CD44 (clone IM7) | BD Bioscience | Cat# 560567; RRID: AB_1727480 |
| APC anti-mouse CD69 (clone H1.2F3) | Biolegend | Cat# 104514; RRID: AB_492843 |
| Purified anti-mouse CD3ε (clone 145-2C1) | BD Bioscience | Cat# 553057; RRID: AB_394590 |
| Purified anti-mouse CD28 (clone 37.51) | BD Bioscience | Cat# 553294; RRID: AB_394763 |
| PE/Cyanine7 anti-mouse IFNγ (clone XMG1.2) | Thermo Fisher | Cat# 25-7311-82; RRID: AB_469680 |
| APC anti-human/mouse granzyme-B (clone QA16A02) | Biolegend | Cat# 372204; RRID: AB_2687028 |
| AF700 anti-mouse TNF-α (clone MP6-XT22) | Biolegend | Cat# 506338; RRID: AB_2562918 |
| PE-Cy7 anti-mouse IL-10 (clone JES5-16E3) | Biolegend | Cat# 505026 ; RRID: AB_11149682 |
| AF488 anti-mouse Ki-67 (clone 16A8) | Biolegend | Cat# 652418; RRID: AB_2564236 |
| PE-Cy7 anti-mouse Foxp3 (clone FJK-16s) | Thermo Fisher | Cat# 25-5773-82; RRID: AB_891552 |
| PE anti-mouse RORγt (clone Q31-378) | BD Bioscience | Cat# 562607; RRID: AB_11153137 |
| APC anti-mouse GATA3 (clone 16E10A23) | Biolegend | Cat# 386908; RRID: AB_3097429 |
| Purified anti-mouse CD16/32 (clone 93) | Biolegend | Cat# 101302; RRID: AB_312801 |
| APC-Cy7 anti-mouse CD11b (clone M1/70) | Biolegend | Cat# 101226; RRID: AB_830641 |
| Percp-Cy5.5 anti-mouse CD11b (clone M1/70) | Biolegend | Cat# 101228; RRID: AB_893232 |
| FITC anti-mouse Ly6C (clone HK1.4) | Biolegend | Cat# 128006; RRID: AB_1186134 |
| APC anti-mouse Ly6G (clone 1A8) | Biolegend | Cat# 127614; RRID: AB_1877163 |
| PE-Cy7 anti-mouse F4/80 (clone BM8) | Biolegend | Cat# 123114; RRID: AB_893478 |
| APC-Cy7 anti-mouse CD11c (clone N418) | Biolegend | Cat# 117324; RRID: AB_830646 |
| PE anti-mouse MHCII (clone M5/114.15.2) | Biolegend | Cat# 107608; RRID: AB_313322 |
| APC-Cy7 anti-mouse CD19 (clone 6D5) | Biolegend | Cat# 115530; RRID: AB_830706 |
| AF647 anti-mouse FAS (clone SA367H8) | Biolegend | Cat# 152620; RRID: AB_2922475 |
| Percp-Cy5.5 anti-mouse GL7 (clone GL7) | Biolegend | Cat# 144610; RRID: AB_2562978 |
| PerCP/Cyanine5.5 anti-mouse PD-L1 (clone 10F.9G2) | Biolegend | Cat# 124334; RRID: AB_2629832 |
| PE anti-mouse Siglec-F (clone  S17007L) | Biolegend | Cat# 155506; RRID: AB_2750235 |
| AF700 anti-mouse CD31 (clone 390) | Biolegend | Cat# 102444; RRID: AB_2819785 |
| PE anti-mouse EpCAM (clone G8.8) | Biolegend | Cat# 118206; RRID: AB_1134172 |
| APC anti-mouse BST2 (clone 927) | Biolegend | Cat# 127016; RRID: AB_1967101 |
| Anti-human BST2 mAb (clone 6A3-Z1-J7) | Zenbio | Cat# 222103 |
| Ultra-LEAF™ Purified anti-mouse BST2 (clone 927) | Biolegend | Cat# 127031; RRID: AB_2819856 |
| Ultra-LEAF™ Purified anti-mouse Ly6G (clone 1A8) | Biolegend | Cat# 127649; RRID: AB_2572001 |
| Anti-Cytokeratin 7 antibody (clone EPR17078) | Abcam | Cat# ab181598 |
| Anti-Yap1 antibody (clone EPR19812) | Abcam | Cat# ab205270 |
| Anti-Myeloperoxidase antibody (clone EPR20257) | Abcam | Cat# ab208670 |
| Anti-TEF-3 antibody (clone N-G2) | Santa Cruz | Cat# sc-101184; RRID: AB_2203086 |
| Anti-PD-1 antibody (Camrelizumab) | Hengrui | Cat# S20190027 |
| Mouse monoclonal anti-β-actin (clone AC-74) | Sigma-Aldrich | Cat# A2228; RRID: AB_476697; 1:5,000 |
| Mouse monoclonal anti-Flag (clone M2) | Sigma-Aldrich | Cat# F3165; RRID: AB_259529; 1:5,000 |
| Rabbit monoclonal anti-HA (clone C29F4) | Cell Signaling Technology | Cat# 3724; RRID: AB_1549585; 1:1,000 |
| Goat anti-rabbit IgG (H+L) secondary antibody, HRP | Thermo Fisher | Cat# 31460; RRID: AB_228341; 1:10,000 |
| Goat anti-mouse IgG (H+L) secondary antibody, HRP | Thermo Fisher | Cat# 31430; RRID: AB_228307; 1:10,000 |
| **Chemicals, peptides and recombinant proteins** | | |
| PMA | Sigma-Aldrich | Cat# P1585 |
| Ionomycin calcium salt | Sigma-Aldrich | Cat# I3909 |
| Brefeldin A | Biolegend | Cat# 420601 |
| Red cell lysis buffer | TIANGEN | Cat# RT122 |
| DNase I | Sigma-Aldrich | Cat# 11284932001 |
| Collagenase II | Sigma-Aldrich | Cat# C6885 |
| DAPI | Sigma-Aldrich | Cat# D9542 |
| Polyethylenimine | Polysciences | Cat# 24765 |
| Advanced DNA RNA Transfection Reagent | Zeta life | Cat# AD600025 |
| Protein A/G plus agarose beads | Santa Cruz Biotechnology | Cat# sc-2003 |
| Protease Inhibitor Cocktail | TargetMol | Cat# C0001 |
| HEPES Solution | Beyotime | Cat# C0215 |
| β-Mercaptoethanol | Sigma-Aldrich | Cat# M3148 |
| **Critical commercial assays** | | |
| CellTiter-Lumi™ Luminescent Cell Viability Assay Kit | Beyotime | Cat# C0065 |
| RNA Isolater Total RNA Extraction Reagent | Vazyme Biotech | Cat# R401-01 |
| qPCR SYBR Green Master Mix (No Rox) | Yeasen | Cat# 11201ES03 |
| Hyperactive pG-MNase CUT&RUN Assay Kit for PCR/qPCR | Vazyme | Cat# HD101-01 |
| LIVE/DEAD Fixable Yellow Dead Cell Stain Kit | Thermo Fisher | Cat# L34968 |
| Fixation/Permeabilization Solution Kit | BD Biosciences | Cat# 554715 |
| Foxp3/Transcription Factor Staining Buffer Set | Thermo Fisher | Cat# 00-5523-00 |
| **Experimental models: cell lines** | | |
| Mouse: AYP cells | This paper | N/A |
| Mouse: AYP-Scramble cells | This paper | N/A |
| Mouse: AYP-sgYap1 cells | This paper | N/A |
| Mouse: AYP-sgBst2 cells | This paper | N/A |
| Mouse: MFC | Cell library, SIBCB | N/A |
| Mouse: YTN16 | Yamamoto et al.^9^ | N/A |
| Human: HEK293FT cells | Biofeng | N/A |
| **Experimental models: organisms/strains** | | |
| Mouse: *Atp4b^Cre-ERT2^* | SMOC | Strain # NM-KI-210118 |
| Mouse: *LSL*-*Yap1^6A/6A^* | This paper | N/A |
| Mouse: *Tp53^fl/f^* | SMOC | Strain # NM-CKO-18005 |
| Mouse: *Rag2^-/-^* | The Jackson laboratory | Strain # 008449 |
| **Software and algorithms** | | |
| GraphPad Prism 8 Software | GraphPad | http://www.graphpad.com |
| FlowJo Software v10 | Tree star | https://www.flowjo.com |
| Seurat V4.3.0 | CRAN | <https://cran.r-project.org/web/packages/> Seurat/index. html |
| SeuratWrappers V0.3.1 | github | https://bioconductor.org/packages/release/bioc/html/GSVA.html |
| batchelor V1.14.1 | Bioconductor | https://www.bioconductor.org/packages/release/bioc/html/batchelor.html |
| Cellchat V1.6.1 | github | https://github.com/sqjin/CellChat |
| inferCNV V0.8.2 | github | https://github.com/broadinstitute/inferCNV |
| Monocle V2.30.1 | github | https://github.com/cole-trapnell-lab/monocle-release |
| SCENIC V0.9.5 | github | https://github.com/aertslab/SCENIC |
| GSVA V1.46.0 | Bioconductor | https://bioconductor.org/packages/release/bioc/html/GSVA.html |
| ggplot2 V3.4.0 | CRAN | https://cran.r-project.org/web/packages/ ggplot2/index.html |
| pheatmap V1.0.12 | CRAN | https://cran.r-project.org/web/packages /pheatmap/index.html |

**Table S3: list of primers for RT-qPCR**

| Gene name | Forward primer | Reverse primer |
| --- | --- | --- |
| *β-actin* | GGCTGTATTCCCCTCCATCG | CCAGTTGGTAACAATGCCATGT |
| *Yap1* | ATGACAACCAATAGTTCCGATCC | CAGGGTGCTTTGGCTGATG |
| *Bst2* | TGTTCGGGGTTACCTTAGTCA | GCAGGAGTTTGCCTGTGTCT |
| *Cyr61* | CTGCGCTAAACAACTCAACGA | GCAGATCCCTTTCAGAGCGG |
| *Ctgf* | GGGCCTCTTCTGCGATTTC | ATCCAGGCAAGTGCATTGGTA |
| Bst2-binding moif1 | ACTCTCTTTGTTCTTGCAT | AGGAACCCCAGAACAACAT |
| Bst2-binding moif2 | ACAGGCACCAGGTGACT | TAGAAGCAAGAGAAACTT |
| Bst2-binding moif3 | ATTCCAGAAGTTTCTCTTG | TGCAGCCTTTCAGTTTCA |
